# Multivariate statistical approach and machine learning for the evaluation of biogeographical ancestry inference in the forensic field

**DOI:** 10.1038/s41598-022-12903-0

**Published:** 2022-05-28

**Authors:** Eugenio Alladio, Brando Poggiali, Giulia Cosenza, Elena Pilli

**Affiliations:** 1 Department of Chemistry, University of Turin, Turin, Italy; 2 Department of Biology, Forensic Molecular Anthropology Laboratory, University of Florence, Florence, Italy; 3 Present Address: Centro Regionale Antidoping e di Tossicologia “A. Bertinaria”, Orbassano, Torino Italy

**Keywords:** Computational biology and bioinformatics, Biological techniques

## Abstract

The biogeographical ancestry (BGA) of a trace or a person/skeleton refers to the component of ethnicity, constituted of biological and cultural elements, that is biologically determined. Nowadays, many individuals are interested in exploring their genealogy, and the capability to distinguish biogeographic information about population groups and subgroups via DNA analysis plays an essential role in several fields such as in forensics. In fact, for investigative and intelligence purposes, it is beneficial to inference the biogeographical origins of perpetrators of crimes or victims of unsolved cold cases when no reference profile from perpetrators or database hits for comparative purposes are available. Current approaches for biogeographical ancestry estimation using SNPs data are usually based on PCA and Structure software. The present study provides an alternative method that involves multivariate data analysis and machine learning strategies to evaluate BGA discriminating power of unknown samples using different commercial panels. Starting from 1000 Genomes project, Simons Genome Diversity Project and Human Genome Diversity Project datasets involving African, American, Asian, European and Oceania individuals, and moving towards further and more geographically restricted populations, powerful multivariate techniques such as Partial Least Squares-Discriminant Analysis (PLS-DA) and machine learning techniques such as XGBoost were employed, and their discriminating power was compared. PLS-DA method provided more robust classifications than XGBoost method, showing that the adopted approach might be an interesting tool for forensic experts to infer BGA information from the DNA profile of unknown individuals, but also highlighting that the commercial forensic panels could be inadequate to discriminate populations at intra-continental level.

## Introduction

Inference of individual biogeographic ancestry plays an essential role in several genetics fields, from population/anthropological studies with the interpretation of genetic admixture in populations or human population expansion, movement, and interaction (e.g.^1^) to medical applications with the evaluation of disease susceptibility (e.g.^2^). Moreover, other disciplines including epidemiology, pharmacogenomics, and forensics, can benefit from biogeographic ancestry testing. In addition, ancestry analysis is of increasing relevance to crime investigations. Identifying perpetrators of crimes or victims of unsolved cold cases by DNA analysis may be hindered by few or no investigative leads and the consequent absence of reference profiles from perpetrators or database hits. In such cases, there is a need for additional genetic information, such as biogeographical ancestry (BGA), that left the trace sample at the crime scene. The best way to assign an individual into a particular population via genetic testing is to use ancestry informative markers (AIMs) –markers characterized by essential differences in allele frequencies between populations^3–5^. As proposed by several studies (for example^6–25^), short tandem repeats (STRs), single nucleotide polymorphisms (SNPs), insertion/deletion polymorphisms (InDels), and microhaplotypes can be used as AIMs for ancestry inference. However, autosomal single nucleotide polymorphisms are the best choices due to their inherent stability, high density of genome-wide distribution, and pronounced frequency variation among populations. Recently, the application of massively parallel sequencing technologies for BGA tool developments allowed the simultaneous analysis of a significant number of SNPs than the SNaPshot-based minisequencing technology as attested by the development for forensics of commercial and non-commercial panels^26–29^. To date, the statistical clustering methods most used for the inference of the biogeographical ancestry of a person or trace relies on are PCA, STRUCTURE^8,30^ and GenoGeographer^31,32^. However, although they provide easy ways to visualize data clustering, these methods are empirical and not adequate for ancestry inference in the forensic field.

In the last decades, analysts have gradually started to take into account all the variables/predictors simultaneously (i.e., in a multivariate way)^33–42^, since this approach allows to extract from the datasets more information than just looking at them in a univariate way, mainly when large amounts of noisy or redundant data occur. One of the attempts to use a multivariate statistical approach to identify clusters of genetically structured populations was described by Jombart et al.^43^ in 2010. In their paper, Discriminant Analysis of Principal Components (DAPC) was applied to simulated data and the performance of their approach was compared to that obtained using STRUCTURE. Multivariate data analysis techniques can be roughly divided into two main categories: (i) pattern recognition techniques; (ii) regression/calibration models. Very concisely, pattern recognition models can be again divided into two categories: (i) unsupervised models (where the information about the a priori classification of each of the individuals/instances/samples under exam is missing); (ii) supervised/classification models (where the a priori classification of each of the instances under exam is known). One of the most known unsupervised methodologies (also known as exploratory analyses) is Principal Components Analysis (PCA)^44^. On the other hand, supervised/classification modeling techniques can present an important family of strategies known as discrimination models, such as Partial Least Squares-Discriminant Analysis (PLS-DA)^45^, that aim to calculate specific boundaries in the multidimensional space that allow separating the different objects within their corresponding classes^46^. Therefore, for the first time, we decided to adopt a multivariate methodology in the present study, such as Partial Least Squares-Discriminant Analysis (PLS-DA) on several SNPs datasets involving instances of different populations showing different BGA. Our main aim was to build robust multivariate models to interpret the results of BGA inference by using different BGA panels that have been developed for this purpose. The PLS-DA approach has been already adopted by Alladio et al*.*^30^ on DNA STRs data to infer the biogeographical ancestry of unknown individuals, and the developed models provided interesting performances in terms of sensitivity, specificity, and accuracy. However, there are no examples of using such machine learning tool on the more informative SNPs data, especially in terms of ancestry, so that the authors decided to extend this approach on a large amount of data and individuals, too.

Simultaneously, a second and very popular supervised learning algorithm named XGBoost (eXtreme Gradient Boosting) was tested on the collected data to evaluate the performance of another machine learning approach and compare its results with PLS-DA. As well as PLS-DA, no example of this approach for BGA inference have been reported in literature dealing with SNPs data.

The BGA panels evaluated in this study are EUROFORGEN Global AIMs SNP (128 AISNPs here, EUROFORGEN)^28^, Verogen® ForenSeq™ DNA Signature Prep Kit (55 AISNPs here, ForenSeq)^27^, MAPlex—Multiplex for the Asia–Pacific (144 AISNPs here, MAPlex)^29^, and Thermo Fisher HID Ion AmpliSeq™ Ancestry Panel (165 AISNPs here, Thermo Fisher)^26^.

## Methods

### Datasets

The SNPs dataset evaluated in this study was composed of 3,557 individuals from 1,000 Genomes project (2,504 individuals from 26 populations)^47^, Simons Genome Diversity Project (SDGP) (279 individuals from 130 populations) (https://www.simonsfoundation.org/simons-genome-diversity-project/) and Human Genome Diversity Project (HGDP) (929 individuals from 54 populations)^48^.

The individuals shared from the three projects were removed.

All the tested multivariate models were calculated in two steps:

- the first models involved the evaluation of the whole data available by evaluating the different BGA categories in the form of “continental” ancestry, such as African, American, Asian (combining Central, East, North, and South Asia populations), European (involving Middle East populations, too), and Oceanian individuals;
- the following models were built on each continent separately (i.e., Asia, Africa, America, Europe, and Oceania by considering the most represented populations (i.e., 80 individuals, at least).

### Multivariate modeling

PCA, PLS-DA, and XGBoost models were applied on the collected data derived from the different BGA panels. The AIMs profile of each individual (instance) was transformed into a row of zeros and ones by using a one-hot encoding strategy developed in the R environment (version 4.0.2)^49^. In detail, for all the tested subjects, a value equal to 1 was reported for the *n* SNP recorded for each specific AIM, while a value equal to 0 was reported for the other available SNPs of the previously cited AIM. Consequently, each instance’s AIMs profile consisted of a string of 0 and 1 (i.e., one-hot encoding). As similarly reported in^30^, PCA and PLS-DA approaches were used to obtain trustworthy and cross-validated models to infer the BGA information of the available instances. The following R packages were exploited for this purpose: *correlationfunnel*^50^*, dplyr*^51^, *ggplot2*^52^, *mdatools*^53^, *mixOmics*^54^, *mlr*^55^, *plotly*^56^*, pls*, *plsVarSel*^57^ and *xgboost*^58^.

Principal components analysis (PCA, also known as *eigenvector analysis*) was preliminarily employed to perform exploratory analyses and dimension reduction studies on the available data^59–61^. It is commonly used to graphically represent the acquired data by evaluating any subgroup or cluster within the instances and assessing the correlation among the collected features^44,62^. Starting from the original dataset (consisting of a matrix X), PCA aims to eliminate redundant and noisy information by selecting a small number of variables, leading to a better understanding of the data structure. PCA calculates new orthogonal (i.e., uncorrelated) variables, named Principal Components (PCs), that represent a linear combination of the original variables aimed to reproduce the structure of the original data (X), but in an optimal and interpretable way. In practice, PCA approach decomposes the original matrix X into the product of two new matrixes, named T and P, plus a matrix of residuals E, as follows:

$$ {\text{X}}_{(n,p)} = {\text{T}}_{(n,f)} \times {\text{P}}_{(f,p)} + {\text{E}}_{(n,p)} $$

where *n* is the number of instances (here, the genotyped subjects), *p* is the number of predictors (features, here the measured AIMs), and *f* represents the number of selected principal components. The first PC is oriented in the direction of the maximum variance. Afterward, the second PC is oriented towards the maximum residual variance, chosen among the infinite orthogonal directions regarding the first component, etcetera. T matrix contains the objects’ coordinates (called *scores*) in the new multivariate space delimited by a *f*-number of PCs (i.e., scores plot). P matrix gives the variable vector coordinates (called *loadings*) in the new multivariate system (i.e., loadings plot). These linear functions are calculated according to specific weighting coefficients representing the linkage between the original variables and the new components. The loadings are the elements of the eigenvector of the variance–covariance matrix of the original X matrix. Each eigenvector has a corresponding eigenvalue that indicates the amount of variance explained (EV) by each PC. In the present study, only the first *f* PCs were selected to account for a specific percentage (around 80% of cumulative explained variance, CEV) of the system’s overall variance.

Since the PCA approach is primarily an exploratory (unsupervised) data analysis approach, further PLS-DA and XGBoost models were calculated to build properly supervised classification algorithms. These models were evaluated to develop tools capable of predicting and inferring the BGA of new (i.e., unknown) samples and individuals, together with classification probabilities and scores. The adoption of classification models in BGA inference should overcome PCA only since supervised models maximize the covariance between the independent variables (i.e., the SNPs data) and the dependent response (i.e., the BGA of the collected individuals). Moreover, the supervised approaches are particularly suitable for forensic tasks like the one examined in this study since they are appropriately made to predict new unknown samples.

Subsequently, PLS-DA was adopted to investigate the covariance between a matrix X of predictors (i.e., the measured AIMs) and the BGA responses included in a matrix Y. In particular, the main point of multivariate classification models like PLS-DA is to investigate the relationships between X and Y and build a model capable of predicting the BGA responses of new samples whose genotypes will be measured in future caseworks. PLS again calculates new components, called *latent variables* (LV), computed by evaluating X and Y matrixes simultaneously. In particular, from a geometric point-of-view, the latent variables represent a slightly rotated version of the Principal Components^63–66^. While PCA maximizes the X matrix variance, the PLS approach iteratively maximizes the covariance between X and Y. For this purpose, the components calculated on Y are rotated to maximize the covariance concerning the components calculated on X. The iterative process ends when no more helpful information can be extracted from X and Y matrices. Briefly, PLS algorithms can be summarized by the following steps:

1. Calculating two matrices E (= X) and F (= Y) whose columns are centered and normalized;
2. Initializing a vector u is with random values before starting the iterative process;
3. Calculating w ~ E^T^u, where w represents the weights (coefficients) relative to X and the symbol ~ means “*to normalize the result of the operation*”, as suggested by^64^;
4. Calculating t ~ Ew, where t represents the new scores of X;
5. Calculating q ~ F^T^t, where q represents the weights (coefficients) relative to Y;
6. Calculating u ~ Fq, where u represents the new scores of Y;
7. If t has not converged, then the iterative algorithm moves back to step 3. Otherwise, if t has converged, a *b* value is computed. This value allows predicting Y from t by following the equation b = t^T^u. Simultaneously, the loadings of X are computed by following the equation p = E^T^t.
8. Finally, the effect of t is subtracted from both E and F matrixes, as follows: E_final_ = E-tp^T^ and F_final_ = F-btc^T^. In particular, the scalar *b* values are represented by a diagonal matrix B.

The sum of squares of X (Y) explained by the latent vector is computed as p^T^p (*b*^2^ for Y), and the percentage of the variance explained (EV) by the PLSR model is obtained by dividing the explained sum of squares by the corresponding total sum of squares^64^. The discriminant version of PLS (PLS-DA) is computed by classifying the objects through X's regression (PLS) and a matrix Y that contains binary responses. In particular, Y consists of *G* columns equal to the number of categories (i.e., BGA classes). Each column contains the class membership information of the corresponding *n* individuals (instances). Since the response is binary (or one-hot encoded), if an instance belongs to a specific *g-*th category, it shows a response equal to 1 within the *g-*th column. Otherwise, its response is coded as 0.

Finally, the XGBoost algorithm, reported in^58^, was tested since it has become a viral classification algorithm, mainly when data are expressed in a one-hot encoded format (i.e., binary data involving only 0 and 1 values). It frequently shows improved performances compared to the well-known supervised classification approaches. It shows several strengths typical of the tree-based algorithms, such as the capability of handling categorical features (in terms of one-hot encoding) and the possibility of making no assumptions about the distributions of the collected data. The main computational weakness of XGBoost algorithm is the necessity of setting many parameters (*hyperparameters*) before obtaining the best (robust and cross-validated) models. In particular, a grid search approach was used in this study to optimize several hyperparameters such as *eta* (i.e., the learning rate, a number between 0 and 1), *gamma* (i.e., the minimum number of splitting for a node, from 0 up to 14), *max_depth* (i.e., a number that indicates how deeply each evaluated tree can grow, from 1 up to 5), *min_child_weight* (i.e., a value defining the level of impurity sustainable for a node, between 1 and 9), *subsample* (i.e., a value describing the proportion of samples to be randomly sampled during the evaluation of each tree, between 0 and 1), *colsample_bytree* (i.e., a number describing the proportion of features selected by each tree, between 0.5 and 1), and *nrounds* (i.e., the number of trees that can be sequentially built within the model^55,67^. XGBoost models were computed on all the available SNPs collected in this study at inter-continental and continental levels. All XGBoost models (as well as the PLS-DA ones) were expressed in terms of sensitivity, specificity, and accuracy. Confusion matrixes and AUC values of the calculated Receiver operating characteristic (ROC) curves were evaluated, too. AUC values equal to 0.5 suggest no discrimination. In contrast, AUC values between 0.7 and 0.8 indicate that the model has acceptable discrimination and AUC values between 0.8 and 0.9 suggest an excellent capacity of discrimination and values equal to or greater than 0.9 indicate outstanding discrimination^68^.

The hyperparameters’ tuning of all the developed models (PLS-DA and, mainly, XGBoost) was performed using a grid search approach and employing a fivefold cross-validation approach with venetian blinds sampling design. The models reported in this study are, therefore, the best we obtained from tuning our models on the data obtained for the different panels (the values of the hyperparameters are not reported). Root Mean Square Error in Cross Validation (RMSECV) was evaluated when building the PCA and PLS-DA models to define the optimal number of components for the developed models.

All experiments were achieved in accordance with relevant guidelines and regulations.

## Results and discussion

### PCA, PLS-DA and XGBoost models at inter-continental level

**Figure 1:**
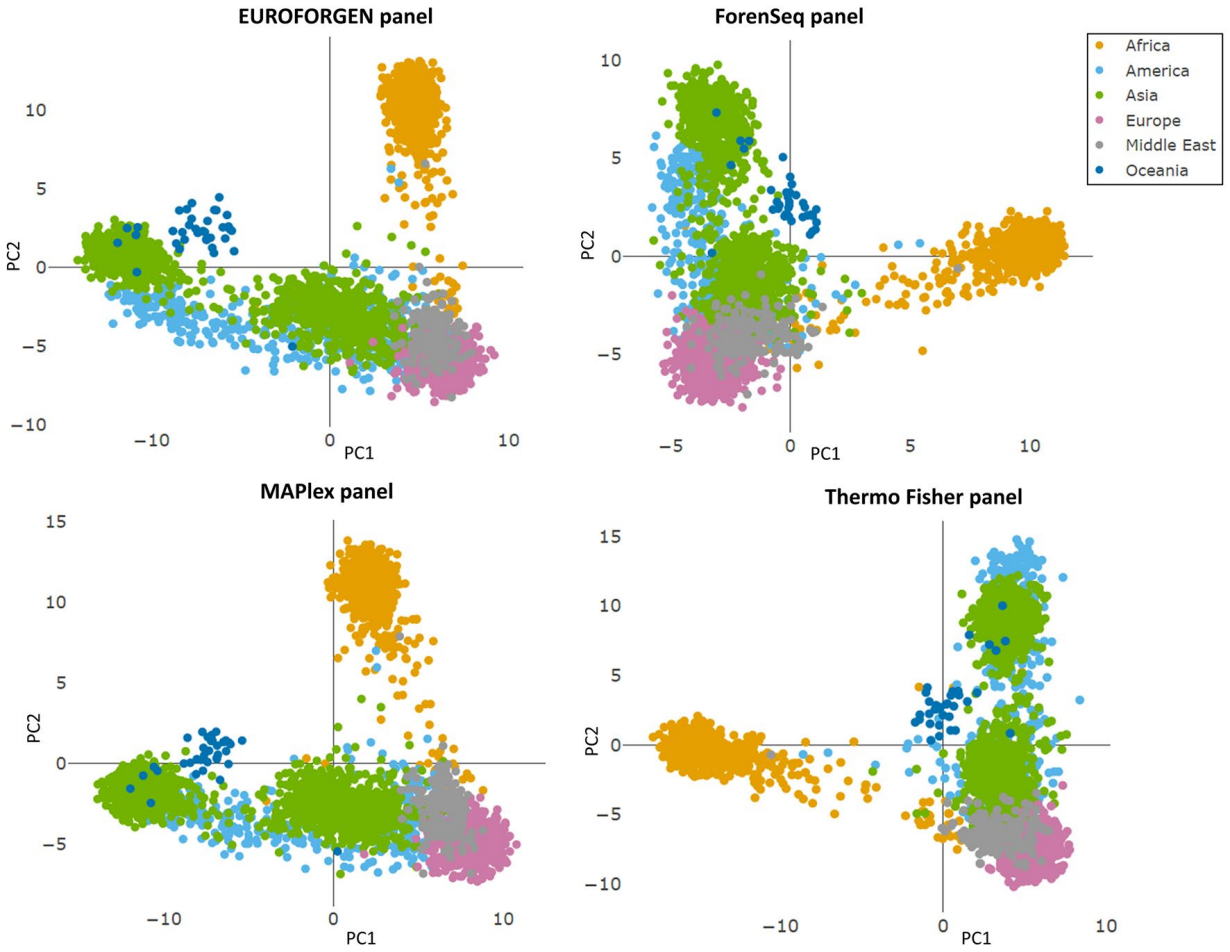
PCA Scores plots showing the PCA models obtained for the different evaluated AIMs panels.

As proposed in different papers^28,69–71^, PCA was first performed to preliminary investigate the available datasets involving the four selected AIMs panels for BGA inference. As expected, for the first level of BGA (i.e., inter-continental BGA) inference, several separate clusters corresponding to African, American, Asian, European, and Oceanian individuals were observed in the space of the first two PCs (Fig. 1). This result turned straightforward for all the evaluated AIMs panels.

After an initial PCA analysis with the Asian continent in its entirety, the Asian was subdivided into its regions due to its breadth -within our dataset, individuals were belonging to different regions of Asia- and the fact that the prediction of the biogeographical origin within the Asian continent has been and is a subject extensively studied in the forensic field^25,72–76^.

As in our dataset, if considering Asia composed by Central, East, North, and South Asia populations, PCA plot highlights that African, East Asian, Oceanian, and (partially) North Asian and European individuals showed a better differentiation from the other tested individuals, while American, South Asian, Central Asian, and Middle East subjects provided an overlap in the PCA space. In addition, better separation of the evaluated populations can be observed in Additional file 1: Fig. S1 also involving three principal components (for a total amount of CEV% equal to 83%).

All the evaluated AIMs panels show a similar degree of separation among individuals belonging to different continental areas. However, only the African individuals reveal a separate cluster in all the panels, presumably due to the history of humans in Africa that is complex and includes demographic events that influenced patterns of genetic variation across the continent, and the fact that modern humans first appeared in Africa roughly 250,000–350,000 years before present and subsequently migrated to other parts of the world^77^.

As shown in Additional file 1: Fig. S1a,b, the African individuals generate an elongated cluster (dark yellow) that extends towards the gray one corresponding to the Middle East region. By evaluating the African individuals closest to the Middle East cluster, we observed that they belong to the populations of northern Africa. The Middle East cluster is in the middle of the European and South Asian clusters and partly overlaps. The light blue cluster that corresponds to the admixed and non-admixed American population is projected toward the European cluster and partly overlaps with it, suggesting that admixed American individuals have an important proportion of European ancestry^78^.

As it can be observed in Fig. 1 and in Additional file 1: Fig. S1, the distribution of the populations in the space of the PCs perfectly reflects the distribution of the populations in the globe: indeed, geographically distant populations are located distantly in the PCA plot, while geographically close populations, regardless of whether they belong to a continent or another, are close in the PCA plot.

Similar PCA plots were obtained by Glusman et al.^79^ and Haber et al.^80^ using a significantly greater number of SNPs, 300,000 and 240,000 respectively than those tested in all the forensic panels. Therefore, as previously highlighted^28,69,70^, despite the limited number of SNPs, the performance of each panel across populations was generally consistent even if some genetic markers performed more than others.

However, although PCA analysis allows us to assign an individual to his/her population of origin through a visual, intuitive, and easy to interpret approach, it does not provide significant divergence between populations, and obviously, it cannot be used alone in forensic context because it does not provide an accurate statistical estimate of the weight of the evidence^69^.

**Figure 2:**
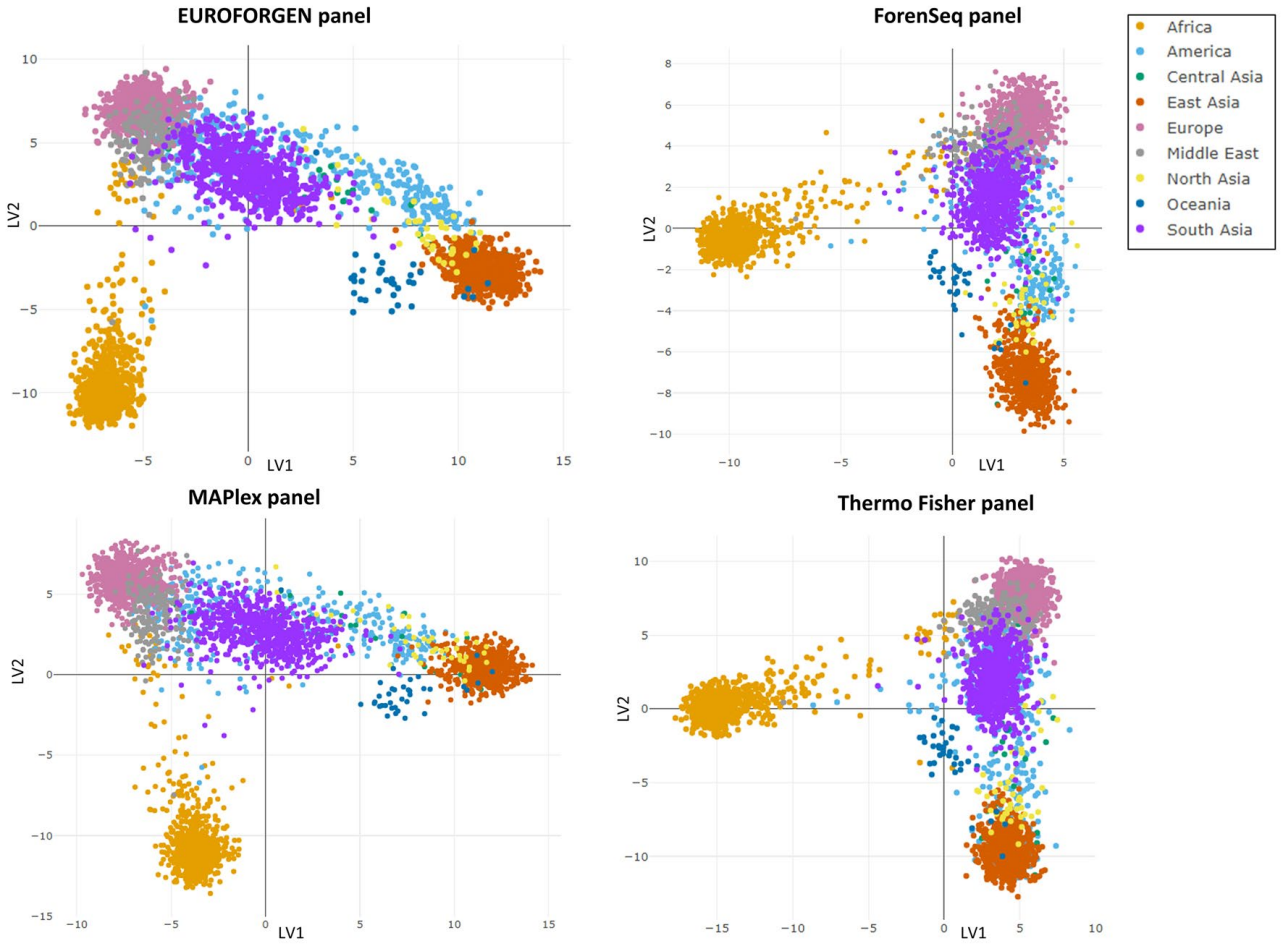
PLS-DA Scores plots showing the models obtained for the different evaluated AIMs panels.

PLS-DA was then applied to the same experimental sets based on PCA modeling results to develop more reliable discrimination models to classify the variables. As a result, for the first level of BGA (i.e., inter-continental BGA) inference, African, American, East Asian, South Asian, Central Asian, North Asian, European, and Oceanian individuals were effectively separated using models involving two latent variables (LVs) (Fig. 2). This result turned noteworthy for all the evaluated panels.

Even if the PCA and PLS-DA plots may seem similar, the obtained Receiver Operating Characteristic (ROC) curves, together with the values of sensitivity, specificity, and AUC highlight the importance of a statistical tool to infer BGA. PLS-DA models for African, American, Asian, European, and Oceanian individuals provided optimal predictions with the CEV% values higher than 98% for all populations in all panels investigated except Oceania—Euroforgen (CEV% 88%), ForenSeq (CEV% 79%), MAPlex (CEV% 86%) and Thermo Fisher (CEV% 79%), and America in ForenSeq (CEV% 95%) panel-. The Oceania population results might be affected by the small number of individuals in the dataset showing this ancestry. All the developed models provided a CEV% higher than 80%, and all the tested AIMs panels proved reliable results that remarked the necessity to use a proper classification model, rather than PCA modeling, to infer BGA robustly.

**Figure 3:**
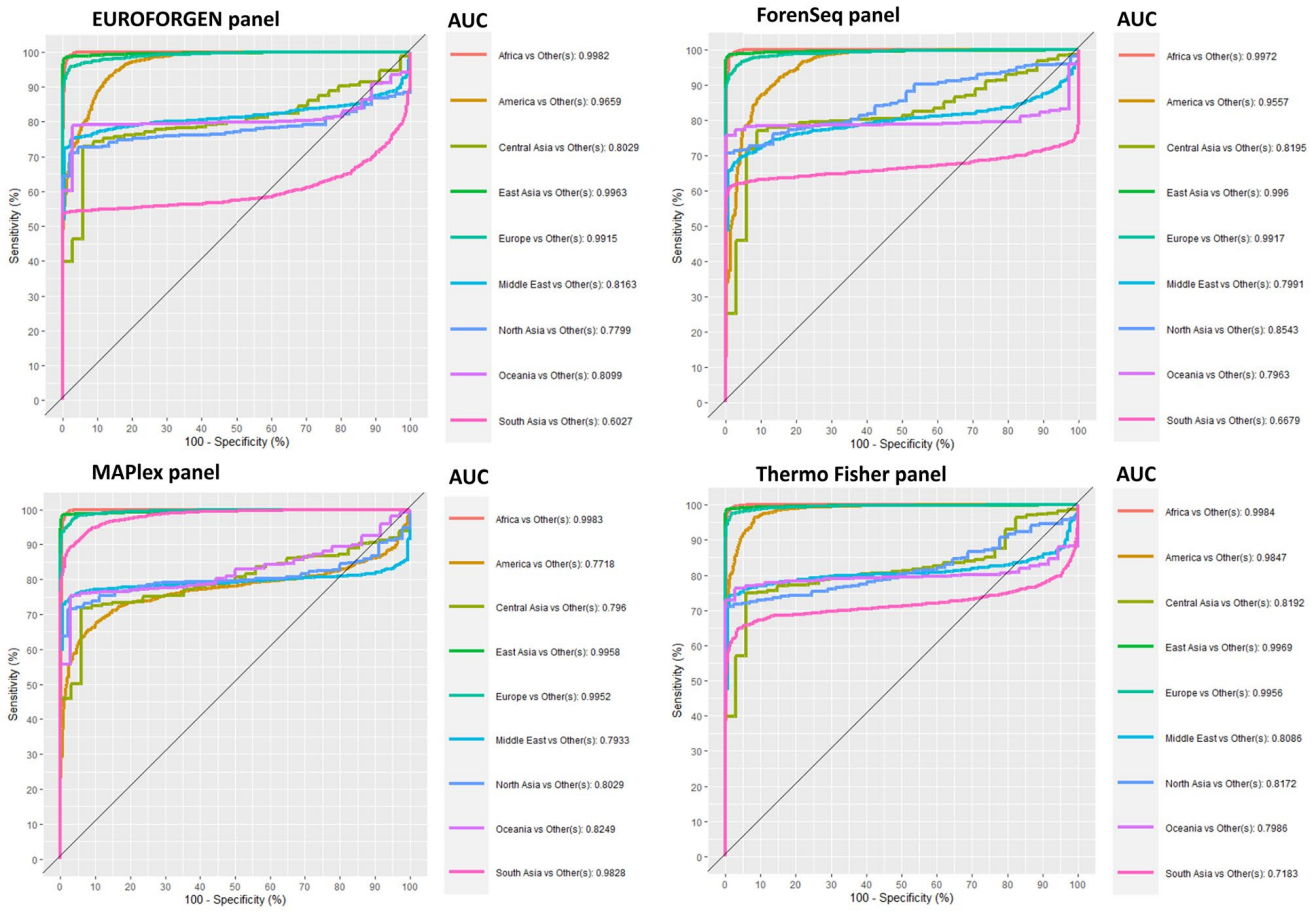
ROC curves, sensitivity, specificity, and AUC values for the tested continental populations.

In addition, through the PLS-DA model, the MaPlex panel ability to differentiate the set of individuals from South Asian to others was estimated with a high degree of accuracy (AUC = 0.9828). As expected from the preliminary assessment of MaPlex^29^, no other panel considered in this study was found to be comparable with it in enhancing South Asian differentiation (Fig. 3). Outstanding discrimination was obtained for East Asian populations in all panels considered associated with less discrimination for Central and North Asian probably due to the limited number of Asian population samples in our dataset, the use of unsuitable markers to discriminate these areas, and the fact that Asia has been a critical hub of human migration and population admixture^81–83^.

As shown in Fig. 3, there are some populations showing poor sensitivity and specificity values. As an example, South Asian individuals have low values for EUROFORGEN, ForenSeq and Thermo Fisher panels, while they are classified with promising results using the MAPlex panel. Similar behaviours are also observed for Middle East and Oceania individuals. These results reflect the fact that some panels, like MAPlex, have been developed to deeply investigate specific populations (i.e., Asia–Pacific populations) and their classification might be prone to better identify such individuals^29^. On the other hand, some populations (like Oceanian and Middle East subjects) showed a lower number of available individuals, compared to the other tested populations, so that the classification performance are not optimal and might be improved by raising the number of investigated subjects.

**Figure 4:**
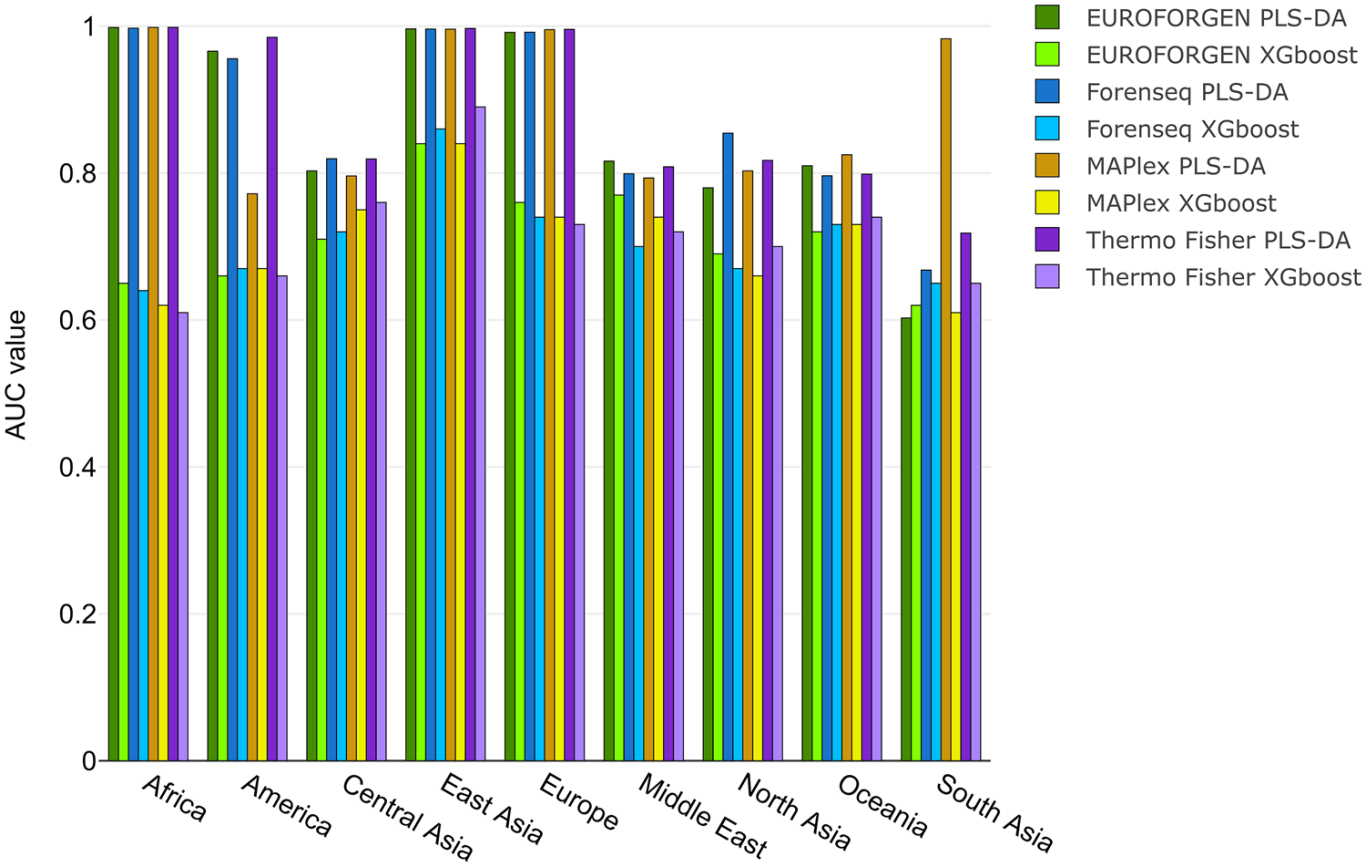
Comparison between AUC values of different populations obtained from PLS-DA and XGBoost model at inter-continental level considering Asian divided into regions.

In accordance with Phillips et al.^29^, our results indicated enhanced South Asian differentiation (AUC = 0.98) using MaPlex panel compared to other forensic panels (Fig. 4), but no increased differentiation between West Eurasian and East Asian populations was detected.

Afterward, the best XGBoost model obtained after the grid search approach provided the following performances (Table 1) in terms of sensitivity, specificity, and AUC. XGBoost algorithm was tested to compare its performances with those from PLS-DA to evaluate another ML model aimed to obtain optimal and feasible inference models for BGA prediction.

**Table 1:** Sensitivity, specificity, and AUC values of the optimal XGBoost model built at inter-continental level for all panels investigated.

Populations	EUROFORGEN			ForenSeq			MAPlex			Thermo Fisher		
	Sensitivity	Specificity	AUC	Sensitivity	Specificity	AUC	Sensitivity	Specificity	AUC	Sensitivity	Specificity	AUC
Africa	0.53	0.77	0.65	0.51	0.78	0.64	0.48	0.76	0.62	0.47	0.76	0.61
America	0.46	0.86	0.66	0.48	0.86	0.67	0.48	0.87	0.67	0.44	0.87	0.66
Central Asia	0.70	0.73	0.71	0.72	0.72	0.72	0.71	0.78	0.75	0.73	0.80	0.76
East Asia	0.78	0.91	0.84	0.83	0.88	0.86	0.79	0.88	0.84	0.84	0.93	0.89
Europe	0.63	0.89	0.76	0.58	0.89	0.74	0.63	0.85	0.74	0.58	0.88	0.73
Middle East	0.72	0.83	0.77	0.62	0.79	0.70	0.68	0.79	0.74	0.71	0.73	0.72
North Asia	0.56	0.82	0.69	0.61	0.73	0.67	0.59	0.74	0.66	0.57	0.82	0.70
Oceania	0.70	0.73	0.72	0.70	0.77	0.73	0.70	0.77	0.73	0.71	0.77	0.74
South Asia	0.50	0.73	0.62	0.55	0.75	0.65	0.49	0.73	0.61	0.55	0.75	0.65

As it can be seen by the values reported in Table 1, XGBoost model provides interesting results, but slightly lower than those of PLS-DA models, especially when comparing the AUC values (Fig. 4).

As shown in Fig. 4, optimal AUC values (close to 1) were observed for African, American, East Asian, and European populations using PLS-DA method, while lower results (around 0.8) were obtained for Central Asia, Middle East, North Asia, Oceania, and South Asia (with the exception of MAPlex panel involving a PLS-DA model) areas. The best results were achieved when using PLS-DA modeling, showing AUC values substantially higher than those obtained by XGBoost. The worst predictions were those involving the South Asian populations overall with AUC values around 0.6. In parallel, STRUCTURE software was tested as a benchmark comparison. The AUC of STRUCTURE was calculated by comparing the ancestry predictions from STRUCTURE software with the real ancestry origins of the tested populations and individuals. Firstly, the number of K clusters (i.e., populations) we selected for our comparison with STRUCTURE was equal to the number of ancestry populations we tested for the different PLS-DA and XGBoost models at inter-continental and inter-continental levels. Then, using CLUMPP together with STRUCTURE, we were able to obtain the Q-matrices containing the membership coefficients for each individual in each cluster. Therefore, each individual was assigned to the ancestry (k-th cluster) showing the highest membership coefficient: this approach allowed us to obtain ROC curves and AUC values for comparing STRUCTURE approach to the predictions and the performance provided by PLS-DA and XGBoost models.

**Figure 5:**
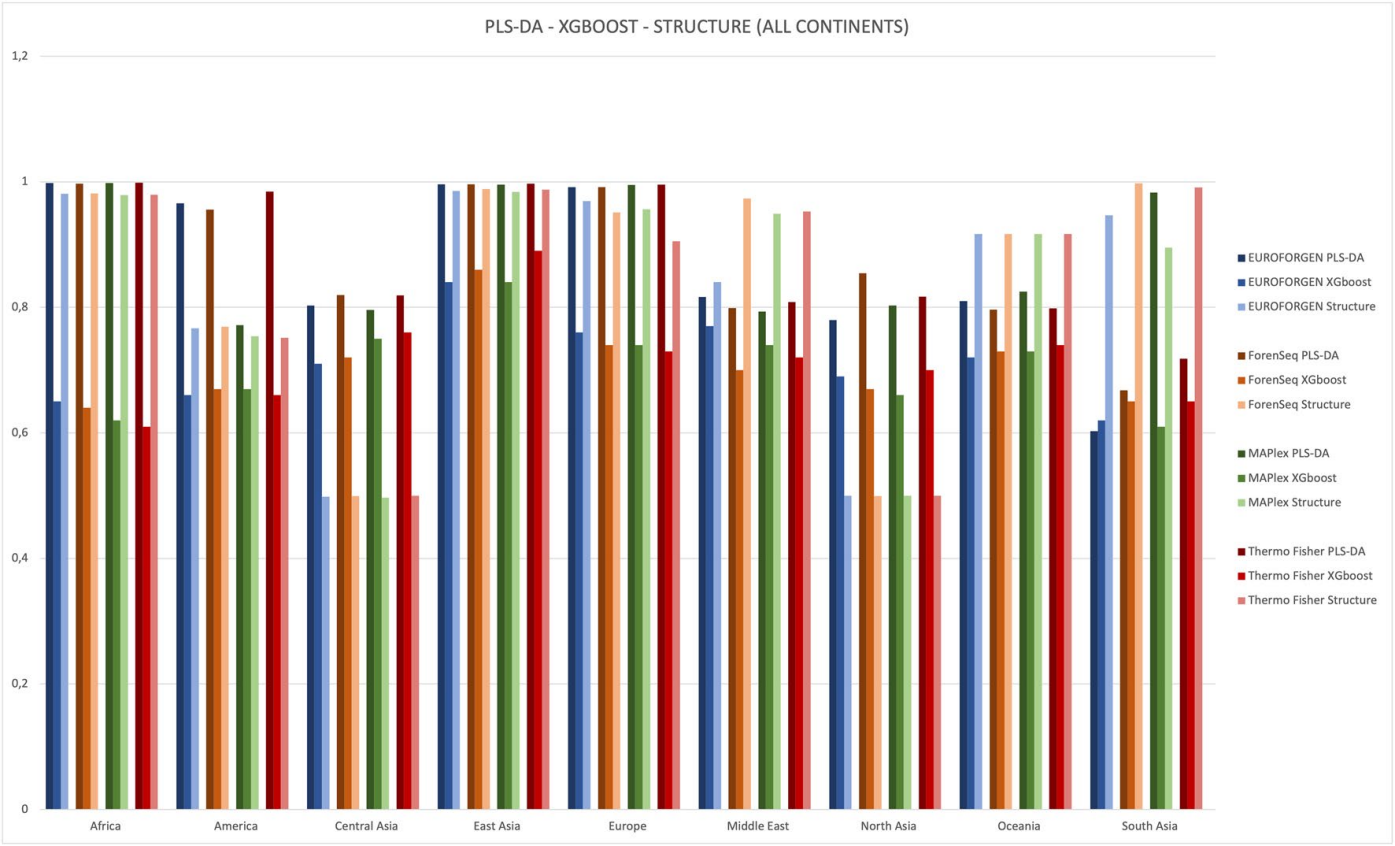
Comparison of AUC values of different populations obtained from PLS-DA, XGBoost, and STRUCTURE at inter-continental level considering Asian divided into regions.

Comparison between AUC values of different populations obtained from PLS-DA, XGBoost and STRUCTURE model at inter-continental level is reported in the Fig. 5. As it can be observed in Fig. 5, better performance was achieved when using PLS-DA modeling rather than STRUCTURE for diverse continents such as Africa, America, Europe and most of Asia (central, east and north Asian) for all panels investigated. Different results were observed in south Asia, Middle East and Oceania where STRUCTURE model seems to work best in almost all panels investigated with the exception of MaPlex panel in South Asia. The worst predictions were those involving XGBoost with AUC values on average lower than STRUCTURE except for Central Asian and North Asia.

### PCA, PLS-DA and XGBoost models at intra-continental level

**Figure 6:**
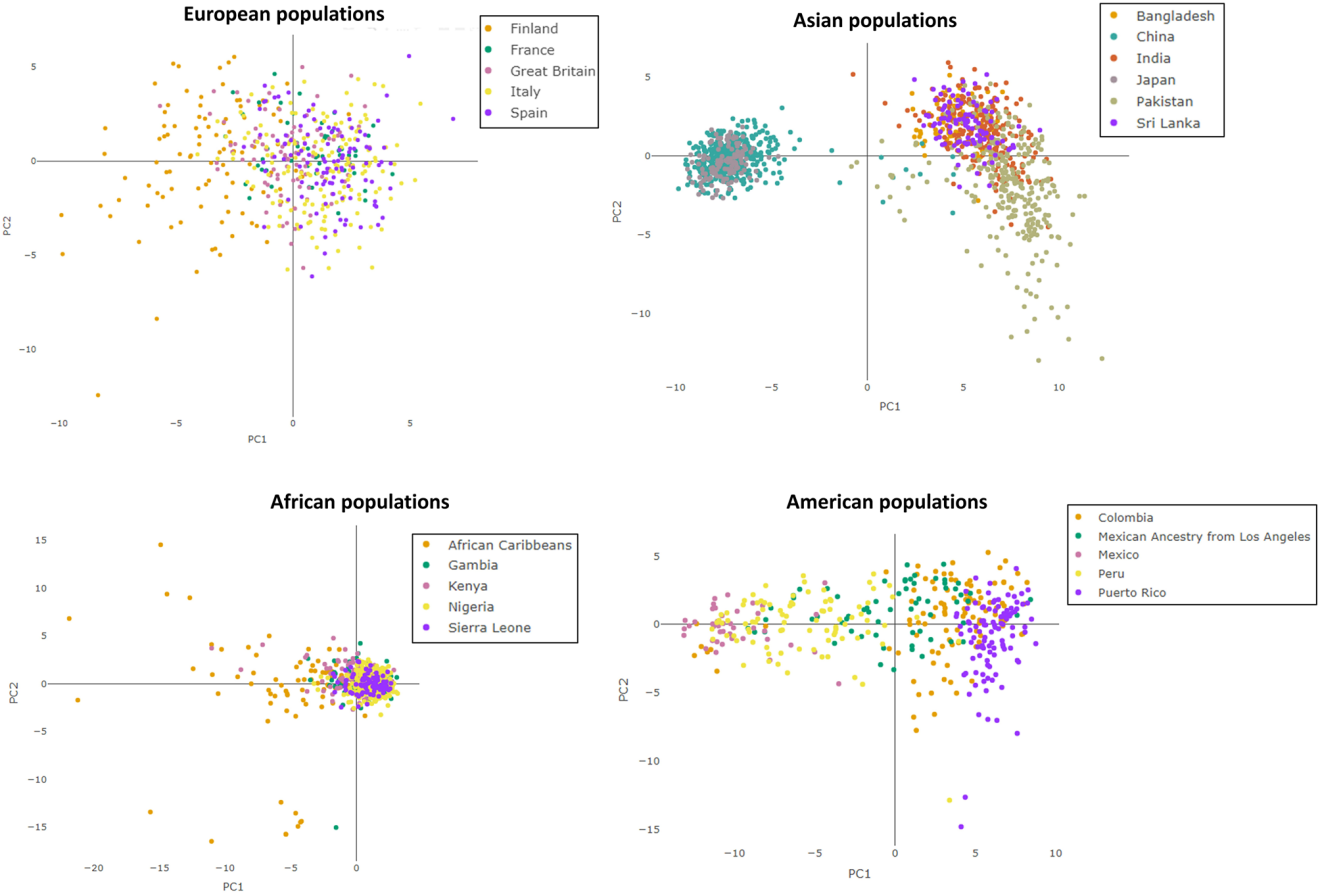
PCA Scores plots showing the PCA models obtained for the different countries and populations tested using the Maplex panel.

PCA model was assessed to infer BGA at continental level and, as expected^28,69^, unsatisfactory separations were observed (an example is shown in Fig. 6 for MAPlex panel). In particular, the following countries and populations were evaluated for the different geographical areas:

- Africa: African Caribbeans, Gambia, Kenya, Nigeria, Sierra Leone;
- America: Colombia, Mexican Ancestry from Los Angeles, Mexico, Peru, Puerto Rico;
- Asia: Bangladesh, China, India, Japan, Pakistan, Sri Lanka;
- Europe: Finland, France, Great Britain, Italy, Spain, Israel.

These countries and populations were selected since they showed more than 80 genotyped individuals in the analyzed dataset; therefore, Oceanian individuals were not considered since the number of genotyped subjects was too limited. As observed in Fig. 6, no significant differences or clusters were detected when using PCA exploratory strategy. Considering Asian population plot, Japan and China provided a different cluster when compared to the other Asian countries but despite the MAPlex panel was specifically developed to provide differentiation of Asian population, can discriminate South from East Asian populations but the sub-populations in these geographical areas cannot be separated from each other. Similar results were observed for all the other BGA AIMs panels (Additional file 1: Figs. S2, S3, S4, S5).

In summary, if this traditional multivariate approach allows us to suggest the BGA of known individuals at the inter-continental level, it fails at intra-continental level, presumably due to the statistical method that is incapable to classify the variables.

Therefore, the application of the PCA model can be considered inadequate for forensic BGA inference goals. For this reason, we adopted proper classification models, such as PLS-DA and XGBoost, to improve our models’ performance and obtain adequate separations among the populations.

**Figure 7:**
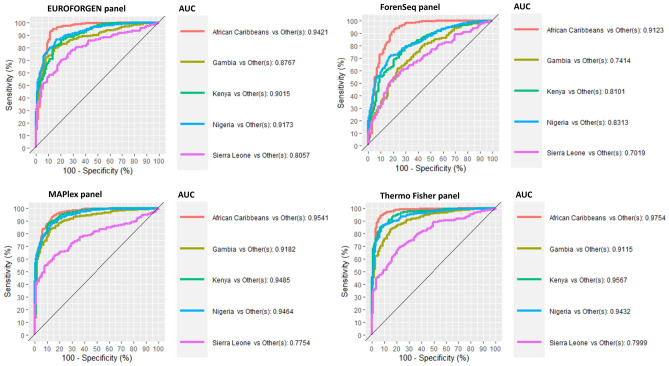
ROC curves, sensitivity, specificity, and AUC values for African countries and populations.

Therefore, PLS-DA and XGBoost models were evaluated at intra-continental level. Figure 7 reports the models and the performance results of the PLS-DA model built to discriminate among the African population.

In the African scenario, the best results were achieved by EUROFORGEN and Thermo Fisher panels, but also MAPlex panel provided interesting results.

The AUC values of the EUROFORGEN panel (Fig. 7) between 0.8 and 0.9 for two out of five populations analyzed and greater than 0.9 for the remaining three, suggest an excellent capacity of discrimination and outstanding discrimination, respectively, of the SNPs in the panel. Thermo Fisher and MaPlex panel obtained similar results.

Presumably, due to the limited numbers of markers in the panel, the worst classification performances were provided by the ForenSeq panel with an average AUC value of 0.798, the lowest value compared to the other panels. These results can also be assessed from the scores plots reported in Additional file 1: Fig. S6 where several clusters are visible from the PLS-DA models built using the different AIMs panels.

The AUC value very close to 100% observed for the African population in all panels tested (Fig. 3) highlights their outstanding discrimination at the inter-continental level and a slightly less capability, albeit excellent in most of the panels, at intra-continental level (Fig. 7). Indeed, the average AUC values for all panels in African population range from an acceptable discrimination for Forenseq panel (average AUC value = 0.798) to an outstanding discrimination for MaPlex and Thermo Fisher panel with the average AUC values equal to 0.92 and 0.91 respectively.

The XGBoost model was also performed, and Tables S1 in Additional file 1 shows the sensitivity, specificity, and AUC values for African populations.

**Figure 8:**
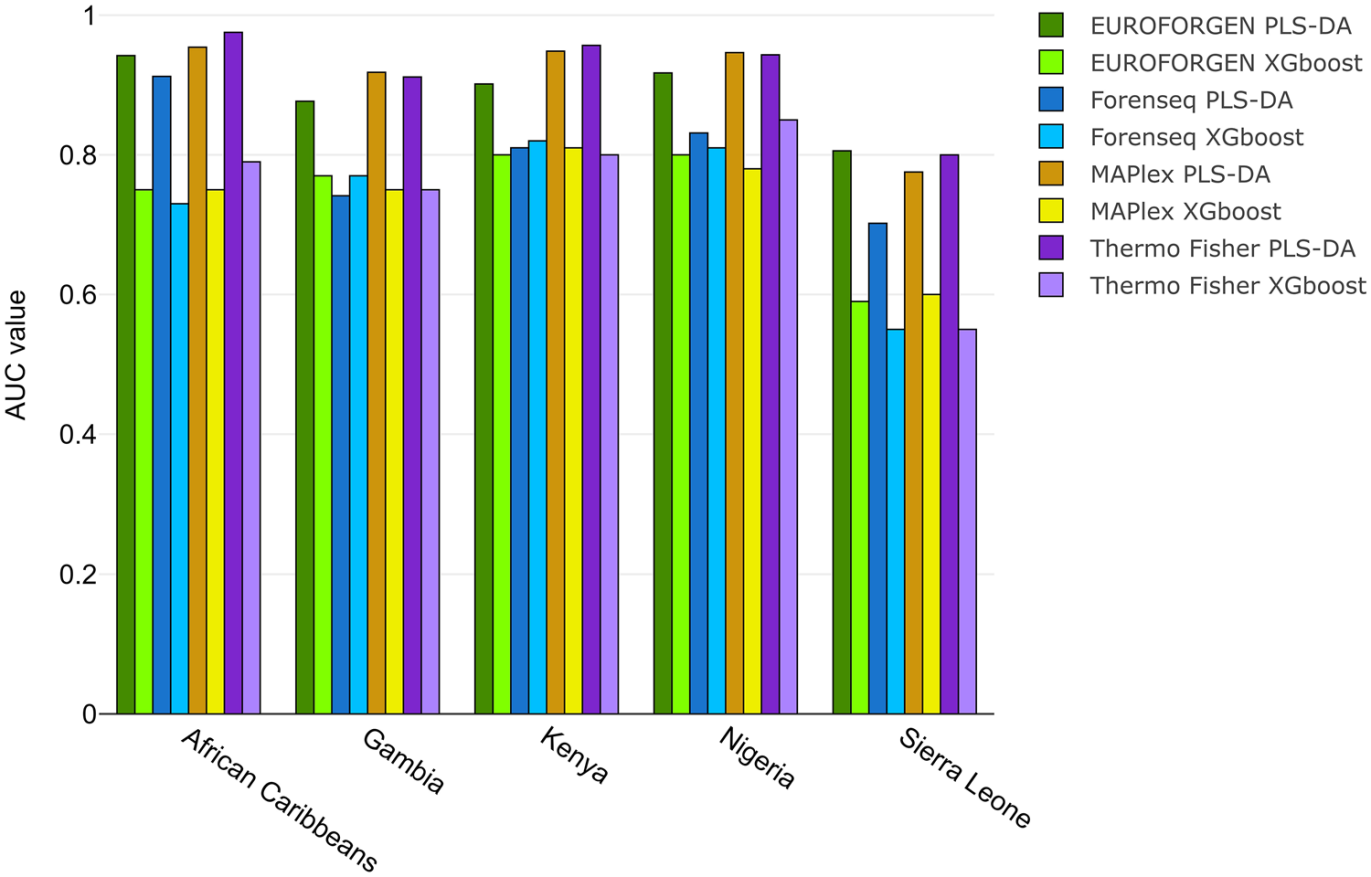
Comparison of AUC values obtained fromPLS-DA and XGBoost model for African population.

AUC values of PLS-DA and XGBoost model were compared (Fig. 8).

Interesting AUC values (around 0.9) were observed for African Carribean, Gambian, Kenyan, and Nigerian individuals, while the worst results (0.8 for PLS-DA, 0.6 for XGBoost) were obtained for the subjects from Sierra Leone presumably influenced by the lower number of individuals in the population. Again, the best performances were achieved using PLS-DA modeling.

**Figure 9:**
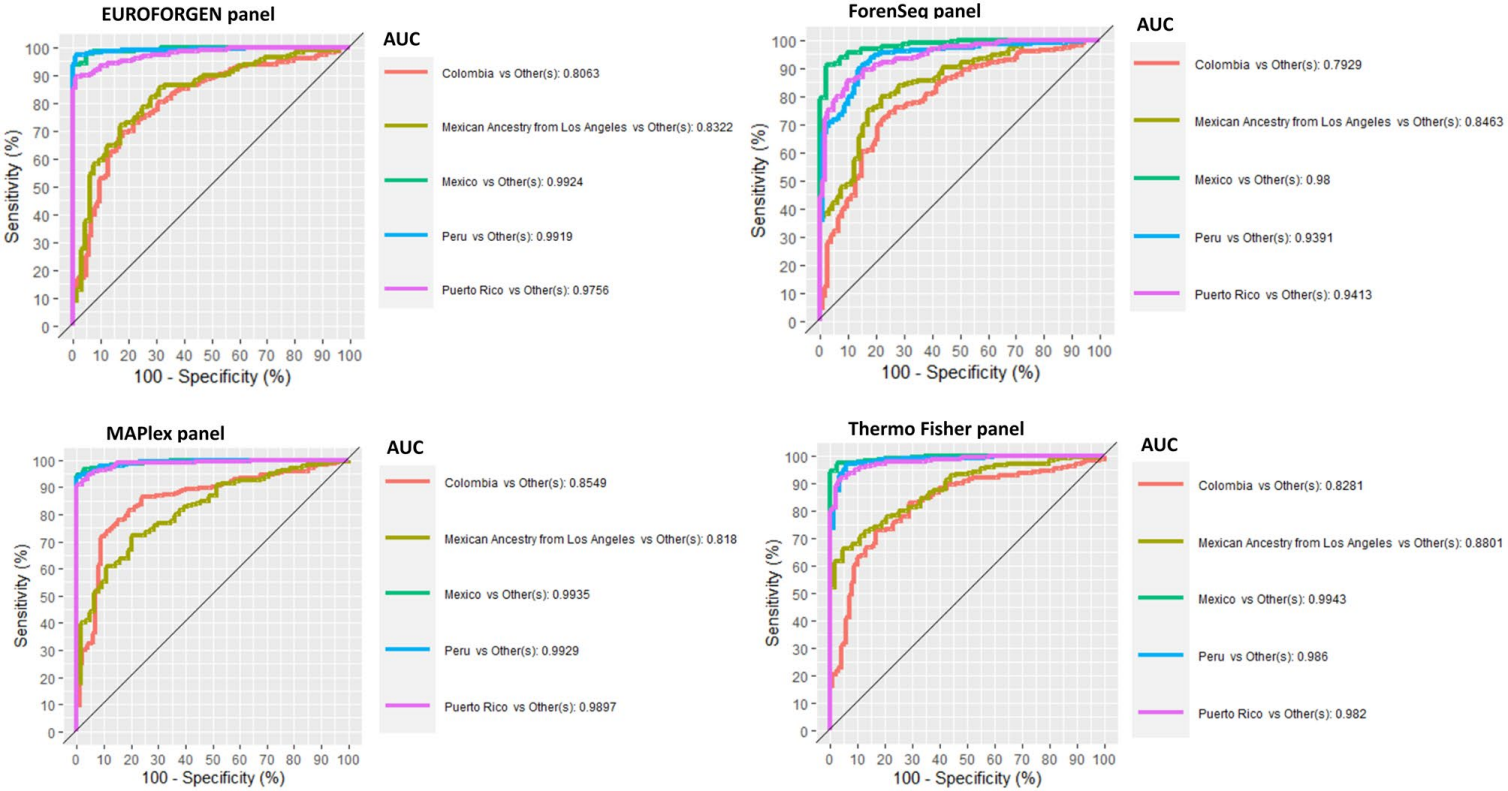
ROC curves, sensitivity, specificity, and AUC values for American countries and populations.

In the American framework (Fig. 9), no specific panel or model outperformed the others. Good discrimination results were observed using EUROFORGEN and MAPlex panels for the individuals from Mexico and Peru, and Puerto Rico (in all cases, AUC value is higher than 0.97), and Colombia (for MAPlex only with an AUC value of 0.85). On the other hand, the Thermo Fisher panel showed the best results in discriminating the individual of Mexican ancestry living in Los Angeles (US) (AUC value of 0.88), but also ForenSeq panel provided remarkable results (AUC value of 0.84). Thermo Fisher panel also provided reliable classification results (AUC value of 0.98) when dealing with subjects from Puerto Rico (as well as EUROFORGEN (0.97) and MAPlex (0.99) panels). These results can also be observed from the scores plots reported in Additional file 1: Fig. S7, showing several clusters among the tested countries and populations.

In addition, in the American scenario, all panels investigated except MaPlex show AUC values higher than 0.95 at inter-continental level (Fig. 3), and a very slightly less capability of discrimination was observed at inter-continental level with the average AUC values higher than 0.90 for all panels (Fig. 9). Therefore, particular attention should be paid with the MaPlex panel. In this case, the AUC value at inter-continental level is much lower (AUC = 0.77) than the average value obtained at intra-continental level (AUC mean = 0.93), showing a better discrimination at intra-continental level rather than at inter-continental one. This might be because there is a lower variability in the analyzed data (as well as in the number of tested populations) and, in this scenario, the algorithms are capable of predicting and inferring BGA with improved performances.

**Figure 10:**
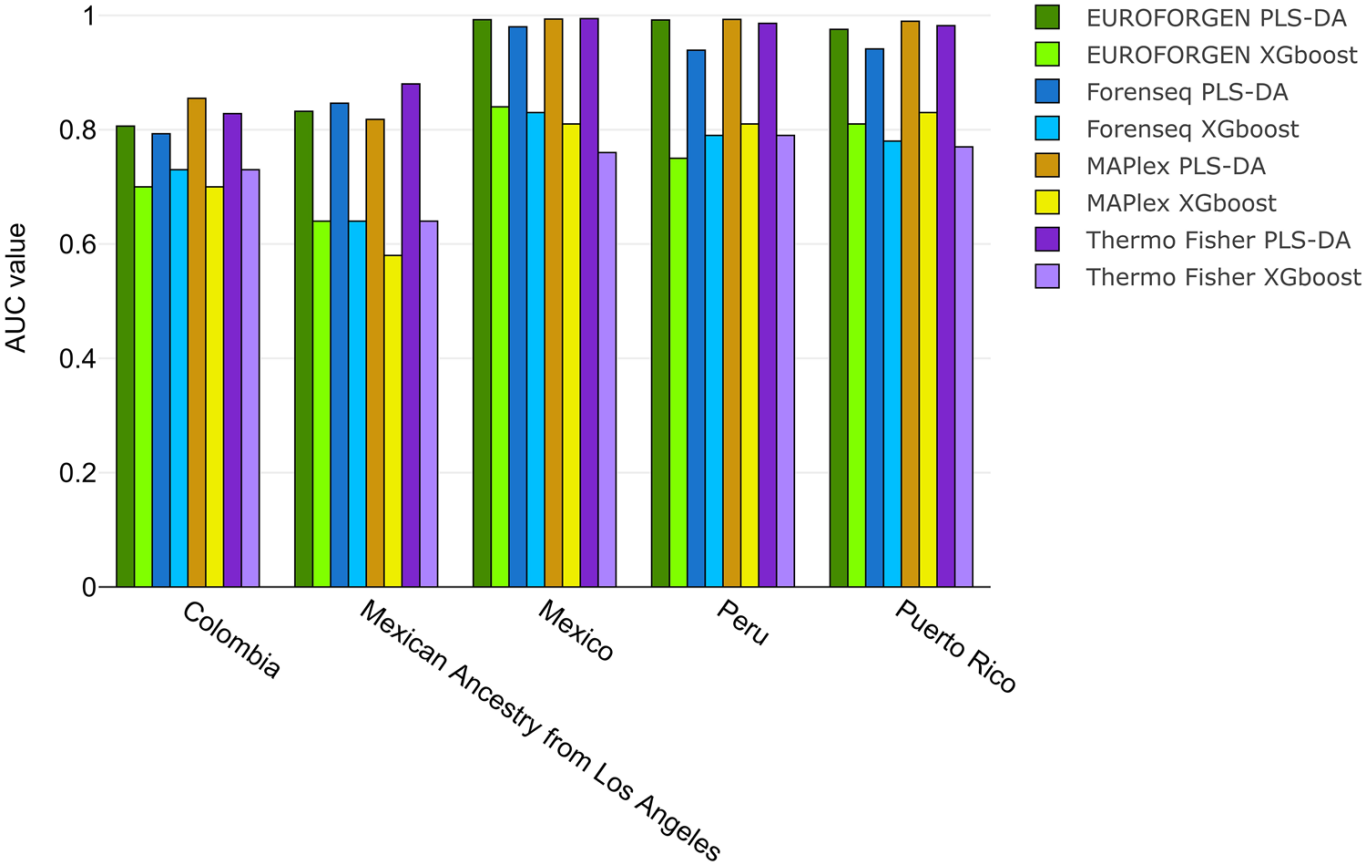
Comparison of AUC values obtained from PLS-DA and XGBoost model for American population.

Tables S2 in additional file 1 shows the sensitivity, specificity, and AUC values of XGBoost model for American population. AUC values of PLS-DA and XGBoost models were compared (Fig. 10).

As shown in Fig. 10, optimal AUC values (around 1 for PLS-DA) were observed when inferring the BGA for individuals from Mexico, Peru, and Puerto Rico, while lower performances (around 0.8 for PLS-DA) were obtained when evaluating Colombian and Mexican Ancestry from Los Angeles individuals. Again, the best performances were achieved using PLS-DA modeling.

**Figure 11:**
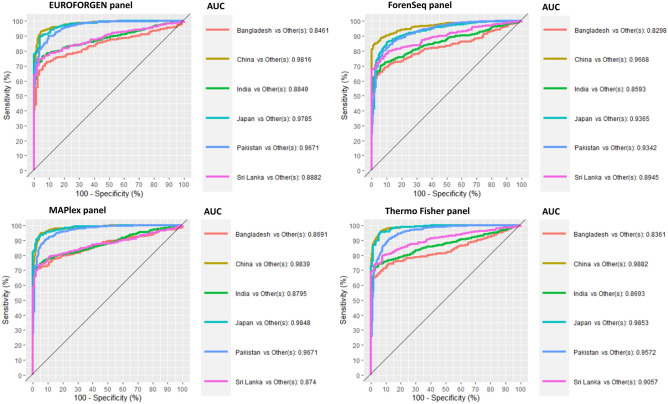
ROC curves, sensitivity, specificity, and AUC values for Asian countries and populations.

In the Asian framework (Fig. 11), similar results were obtained. On average, the best results were obtained when evaluating the Thermo Fisher and MAPlex panels, especially for the individuals from China, Japan, and Pakistan with AUC values equal to 0.99, 0.98 and 0.95, respectively, for Thermo panel and 0.98, 0.98 and 0.86 for MaPlex panel. Excellent discrimination was achieved also for India, Bangladesh and Sri Lanka with AUC greater than 0.80, showing the ability of these two panels to differentiate sub-populations.

The scores plot provided two separated clusters; the first one consists of China and Japan, while the second cluster reported the individuals from Bangladesh, India, Pakistan, and Sri Lanka (Additional file 1: Fig. S8).

**Figure 12:**
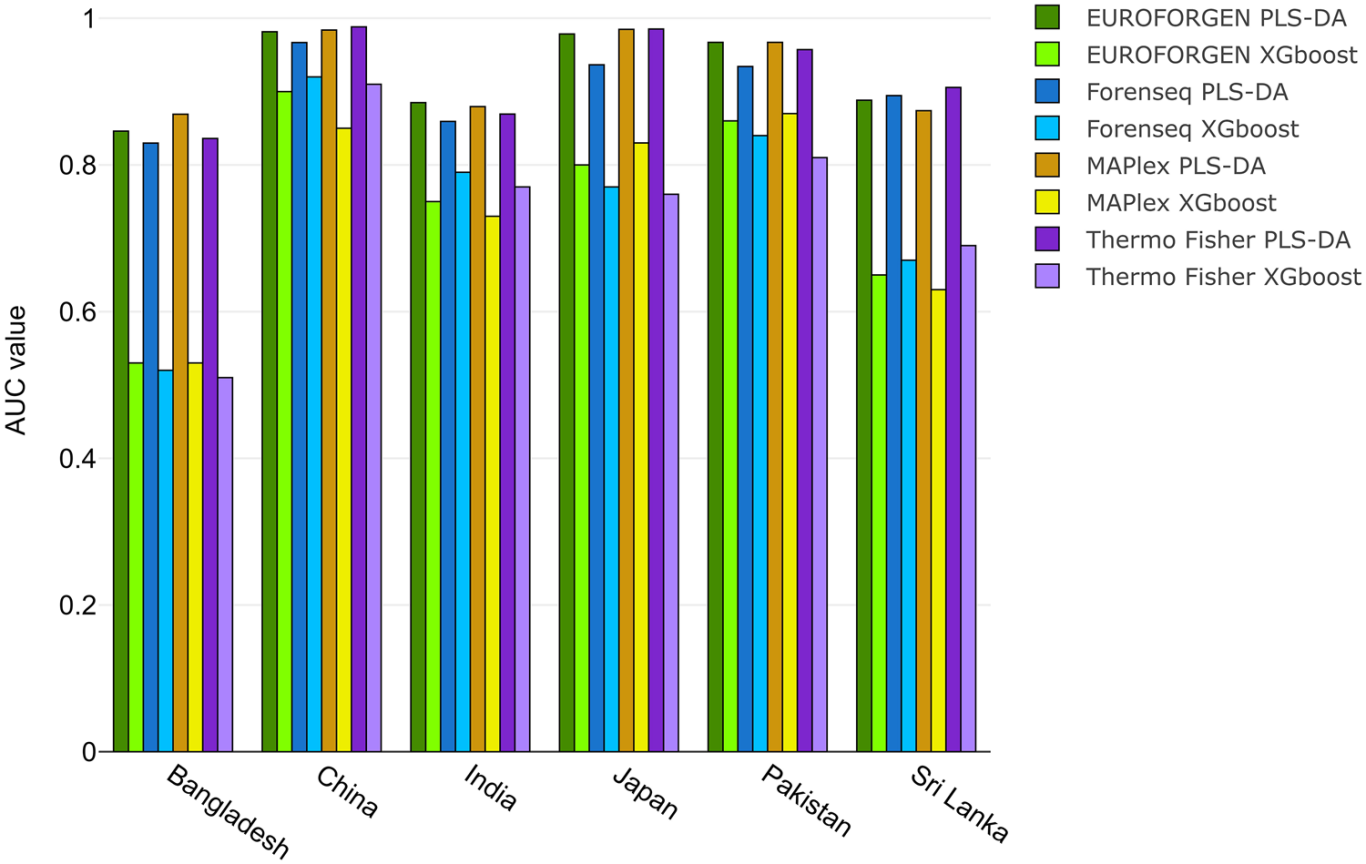
Comparison of AUC values obtained from PLS-DA and XGBoost model for Asian population.

Tables S3 in additional file 1 shows the sensitivity, specificity, and AUC values of XGBoost model for Asian population. AUC values of PLS-DA and XGBoost models were compared (Fig. 12).

The best AUC values (around 1 for PLS-DA) were obtained when inferring the BGA for individuals from China, Japan, and Puerto Rico, while lower results (around 0.8 for PLS-DA) were obtained when evaluating individuals from Bangladesh, India, and Sri Lanka. The lowest results were showed by the XGBoost model on Bangladesh subjects and, once again, the best performances were achieved with PLS-DA modeling.

Finally, no specific AIMs panel or model outperformed the others when evaluating the European countries and populations except for the ForenSeq panel that presents the worst results, presumably due to the low numbers of markers analyzed. The scores plot provided several separate clusters for all the evaluated populations, and these results can also be observed from the scores plots reported in Additional file 1: Fig. S9.

**Figure 13:**
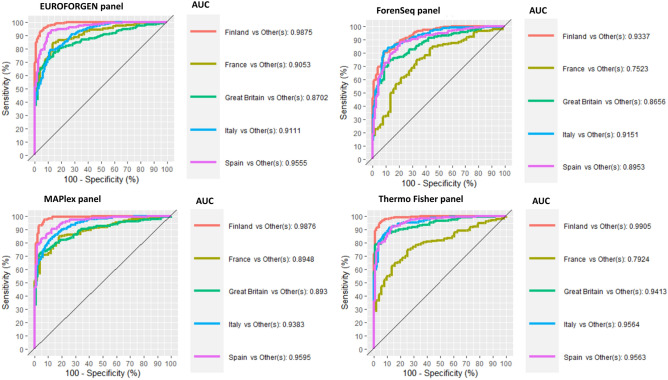
ROC curves, sensitivity, specificity, and AUC values for European countries and populations.

As shown in Fig. 13, the best discrimination result was achieved for Finland populations (AUC ≥ 0.93) for all panels investigated. It has to be noted that the best results for the French individuals were obtained with EUROFORGEN and MAPlex AIMs panels, while for the other groups (Italians, English, Spanish, and Finns) the results are comparable.

**Figure 14:**
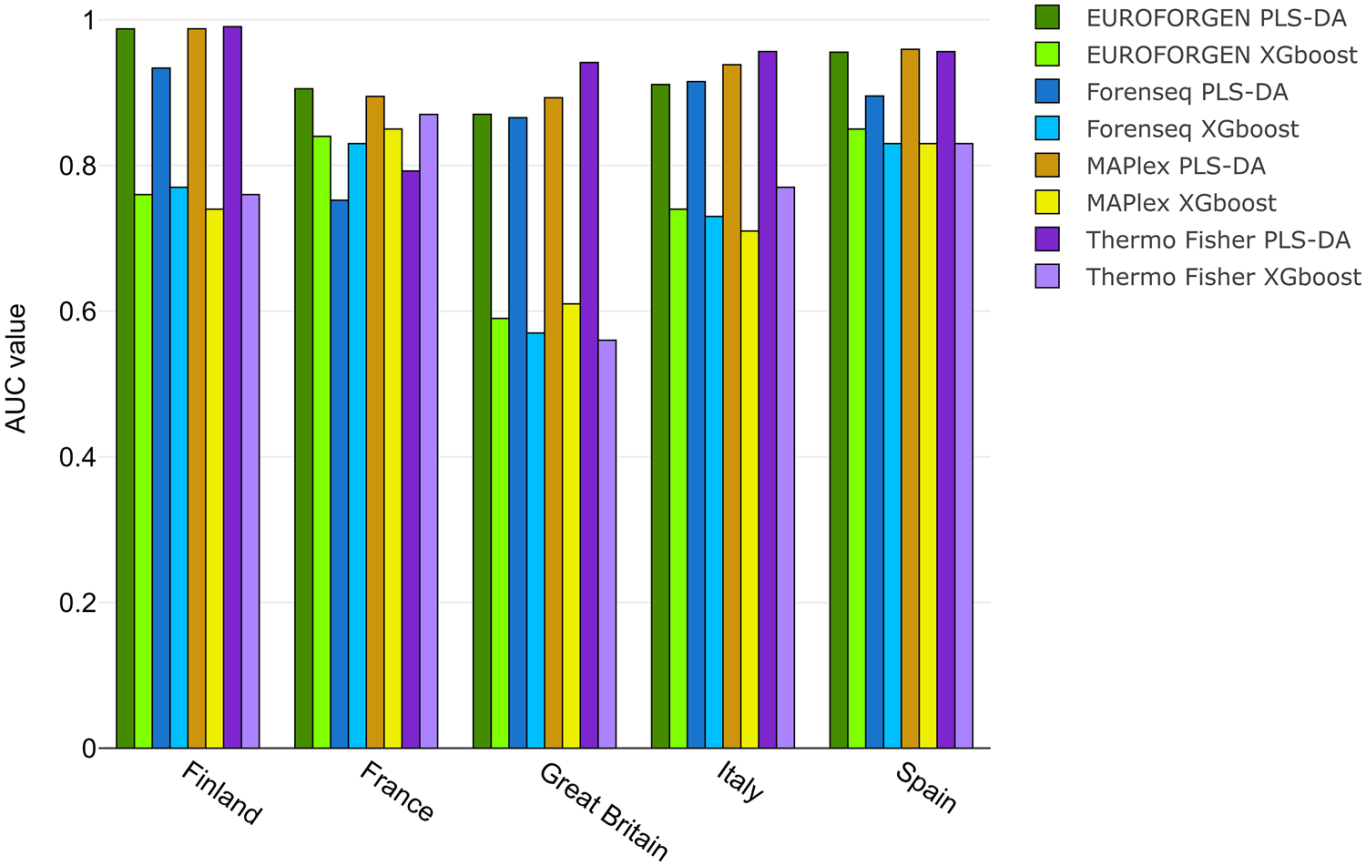
Comparison of AUC values obtained from PLS-DA and XGBoost model for European population.

Tables S4 in additional file 1 shows the sensitivity, specificity, and AUC values of XGBoost model for European population. AUC values of PLS-DA and XGBoost models were compared (Fig. 14).

Optimal AUC values (around 0.9–1) were observed for all the PLS-DA models in this scenario, instead of the XGBoost models showing significantly lower results.

**Figure 15:**
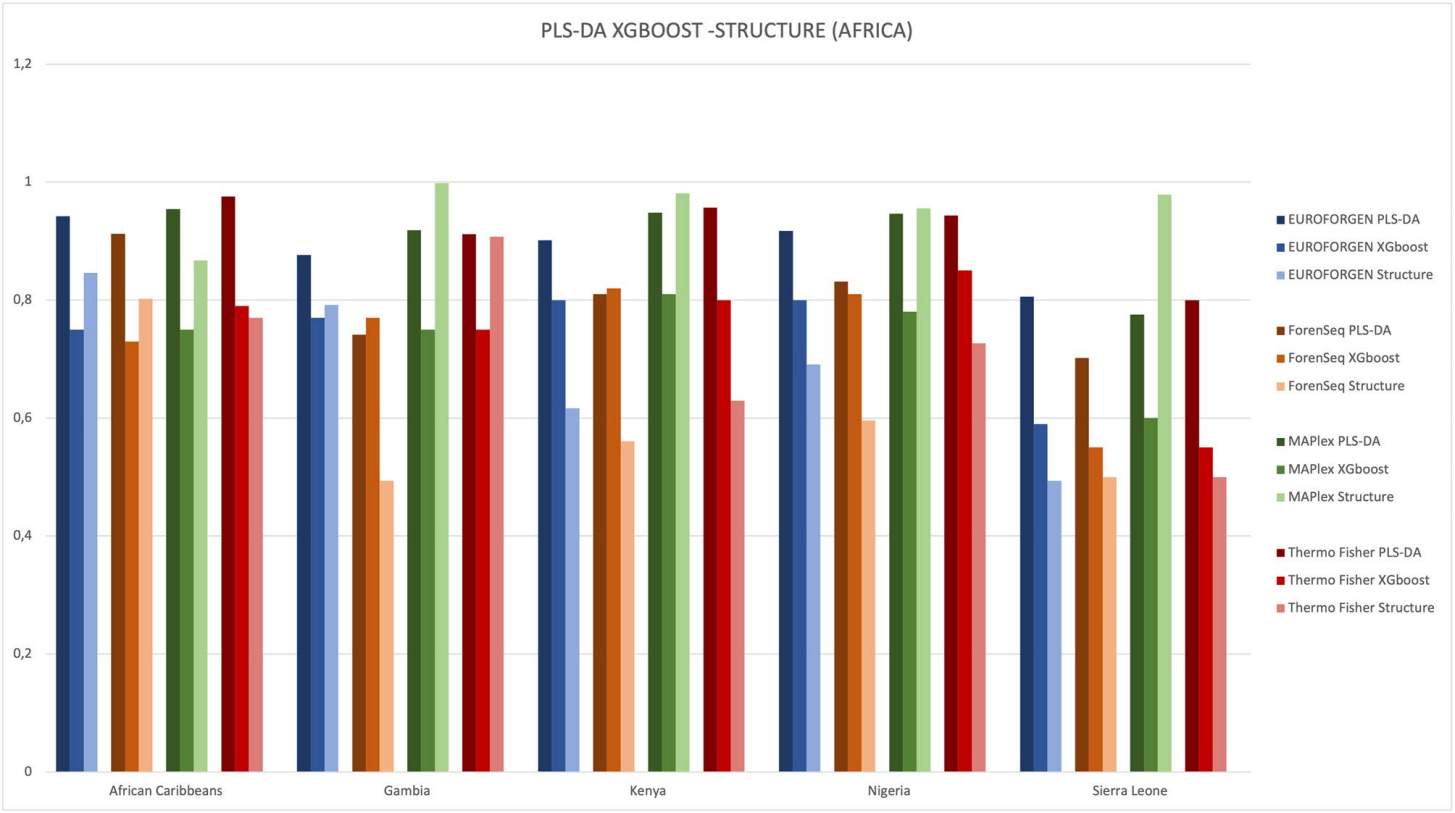
Comparison of AUC values obtained from PLS-DA, XGBoost and STRUCTURE model for African population.

STRUCTURE approach was also compared with PLS-DA and XGBoost model at intra-continental level by evaluating the populations selected for the Africans, as an example. The results in terms of comparison of the AUC values are reported in the Fig. 15. As already observed at inter-continental level, PLS-DA, and in most cases XGBoost, provided, on average, better performance in terms of accuracy when compared to STRUCTURE approach also at intra-continental level.

Comparing the ROC curves of all forensic panels both at inter-continental level and at intra-continental level, a decrease in the accuracy in inferring BGA at intra-continental level was observed. This decrease may be explained by the natural geographical distribution of some populations: “populations that share geographical borders and cultural practices are closely related genetically and these populations show similar genetic patterns”^72^, by the SNPs in forensic panels, selected with the aim of discriminating populations at continental level^28,69,73^, and by their number which is relatively low compared to that used in other genetic fields through NGS technology.

PLS-DA and XGBoost at intra-continental level provided, on average, better performance in terms of accuracy when compared to STRUCTURE approach. In particular, the obtained results showed that PLS-DA performed better than STRUCTURE at both inter- and intra-continental level. Similar results were achieved by Jombart et al.^43^ when using a supervised classification approach like DAPC in comparison with STRUCTURE. Furthermore, both PLS-DA and STRUCTURE methods provide graphical outputs for interpreting the results of the obtained classification models. STRUCTURE provides the results in form of bar plot (being extremely helpful, for instance, when interpreting admixtures) while PLS-DA modelling shows a scatter plot for the tested populations, aimed to evaluate the goodness of the developed classification and allowing to project new individuals into the calculated Scores plots. On the other hand, GenoGeographer approach shows a brilliant use of Likelihood Ratio modelling, since it allows to compare the tested populations and the predictions in terms of Log_10_LR. Similarly, our XGBoost and PLS-DA approaches provide numerical results for the performance of the models (in terms of ROC curves) and the classifications of new individuals (in terms of probability of classification for the new tested individuals).

## Conclusions

Ancestry analysis is of increasing relevance to crime investigations in all situations in which few or no investigative leads are available and genetic information about the donor of the trace or skeleton found at the crime-scene could be of help to police investigations to find unknown perpetrators of crime or identify missing persons. Therefore, the present study investigated the application of multivariate data analysis modelling to discriminate and predict the BGA of several populations by evaluating the AIMs markers and panels available in the market for forensic purposes. PLS-DA and XGBoost supervised models drastically improved the traditionally used PCA approach, by supplying satisfactory classification results and showing a capability of BGA discrimination of the diverse forensic panels. Moreover, the comparison between these models with STRUCTURE approach highlighted a better BGA prediction of PLS-DA than STRUCTURE at both inter- and intra-continental level. Different results were observed at inter-continental level only in south Asia, Middle East, and Oceania where STRUCTURE model seems to work best in almost all panels investigated with the exception of MaPlex panel in South Asia. Despite the high classification performances of PLS-DA, a decrease in the accuracy in inferring BGA was observed for all panels investigated when moving to more geographically restricted populations presumably due to the type of selected SNPs and their limited number in all forensic panels. In addition, particular attention should be paid to the database. Since the analysis of ancestry inference is performed by comparing the sample genotype with one or more known reference population groups, well-characterized databases with high-quality genotyping results of well-defined reference populations are critical. This work represents a proof-of-concept study suggesting the possibility to use supervised algorithms such as PLS-DA or XGBoost (and, eventually, other multivariate models) as a tool for the investigative police forces to estimate the BGA of suspects and persons of interests. Despite the findings, our work does not aim to suggest the use of PLS-DA and XGBoost as improved alternative methods to those involving likelihood-ratio computations and further investigations will be conducted to fully investigate the performance of these approaches and their use for forensic purposes. Future perspective will involve the evaluation of class-modelling ML approaches like SIMCA (Soft-Independent Model of Class Analogy) and the computation of likelihood ratios for each classification; these approaches will allow the forensic expert to obtain interesting information to interpret the results of critical unknown individuals such as admixtures and the cases where AIMs profiles do not belong to any of the included reference populations.

## Supplementary Information

Supplementary Information.

## Data Availability

All data generated or analysed during this study are included in this article and its supplementary information files.
