# Supplementary material for: Multivariate statistical approach and machine learning for the evaluation of biogeographical ancestry inference in the forensic field
Source: Sci Rep. 2022 May 28;12:8974. doi: 10.1038/s41598-022-12903-0 (PMC9148302; doi:10.1038/s41598-022-12903-0)
Supplement: Supplementary file 1 — Supplementary Information. [file 41598_2022_12903_MOESM1_ESM.docx]

**Supplementary materials**


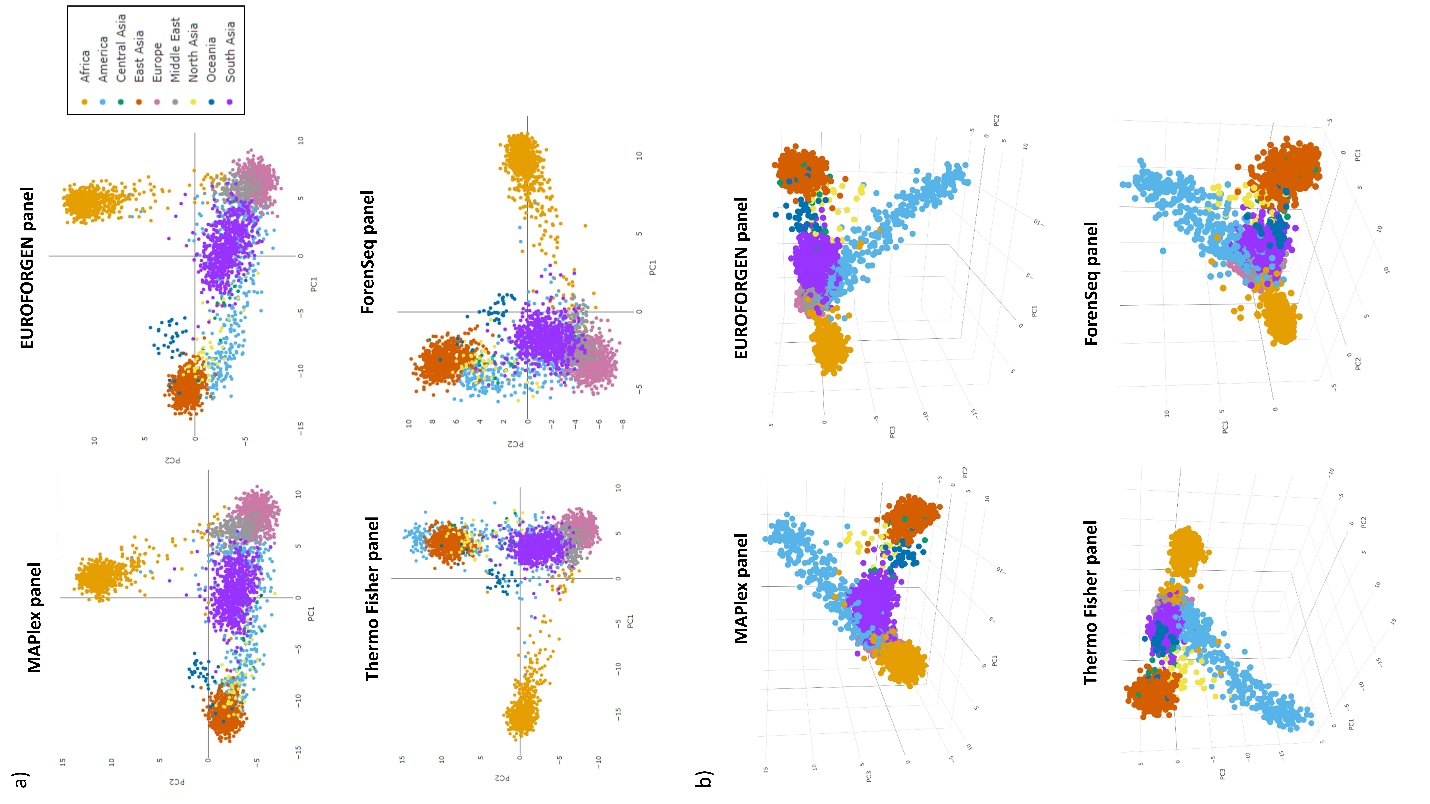


**Figure S.1:** a. PCA Scores plots showing the PCA models obtained for the different evaluated AIMs panels considering Asia composed by Central, East, North, and South Asia populations; b. PCA Scores plots involving 3 PCs and showing the PCA models obtained for the different evaluated AIMs panels.


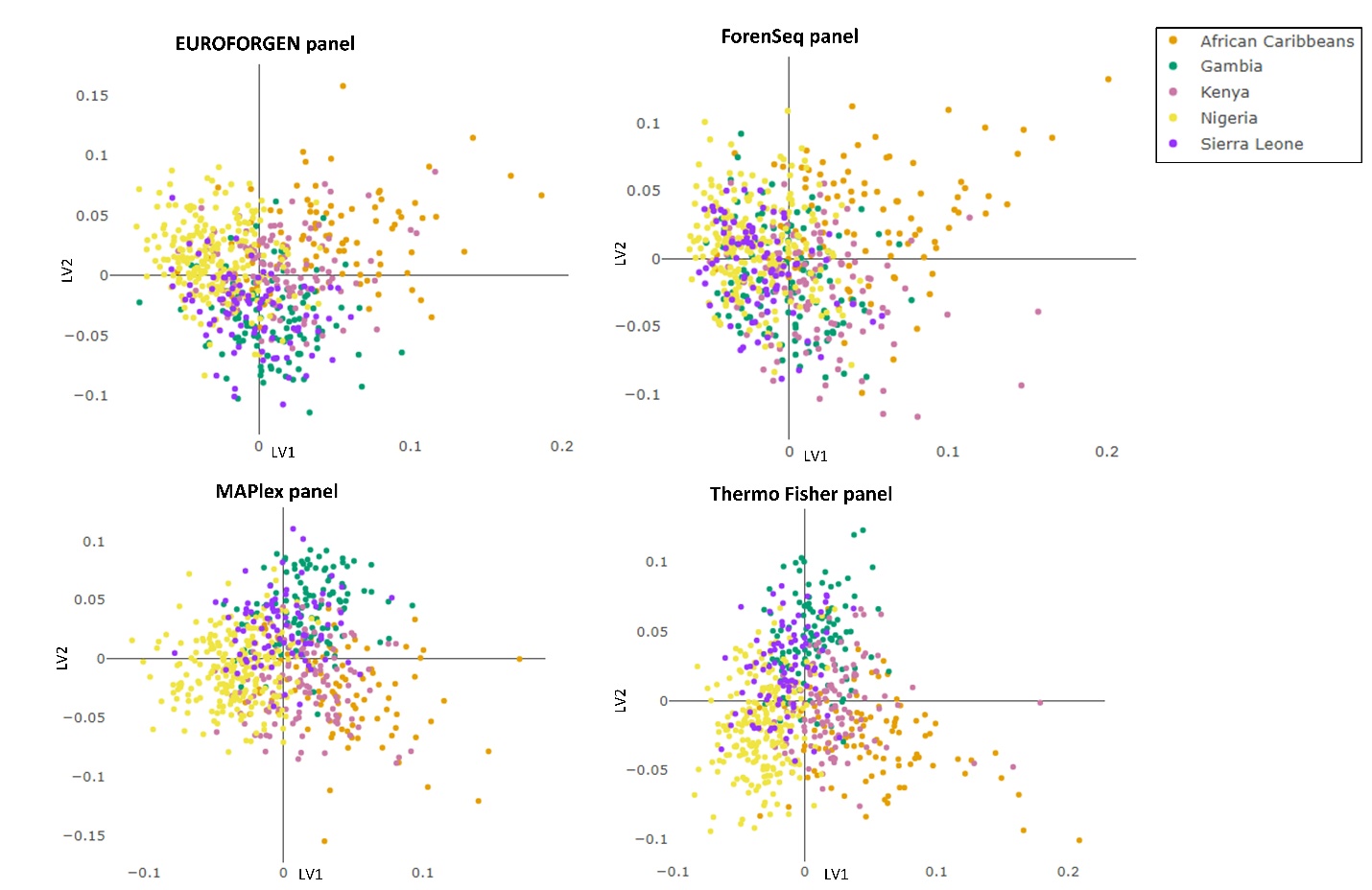


**Figures S.2:** PCA Scores plots showing the PCA models obtained for the African population using all forensic panels


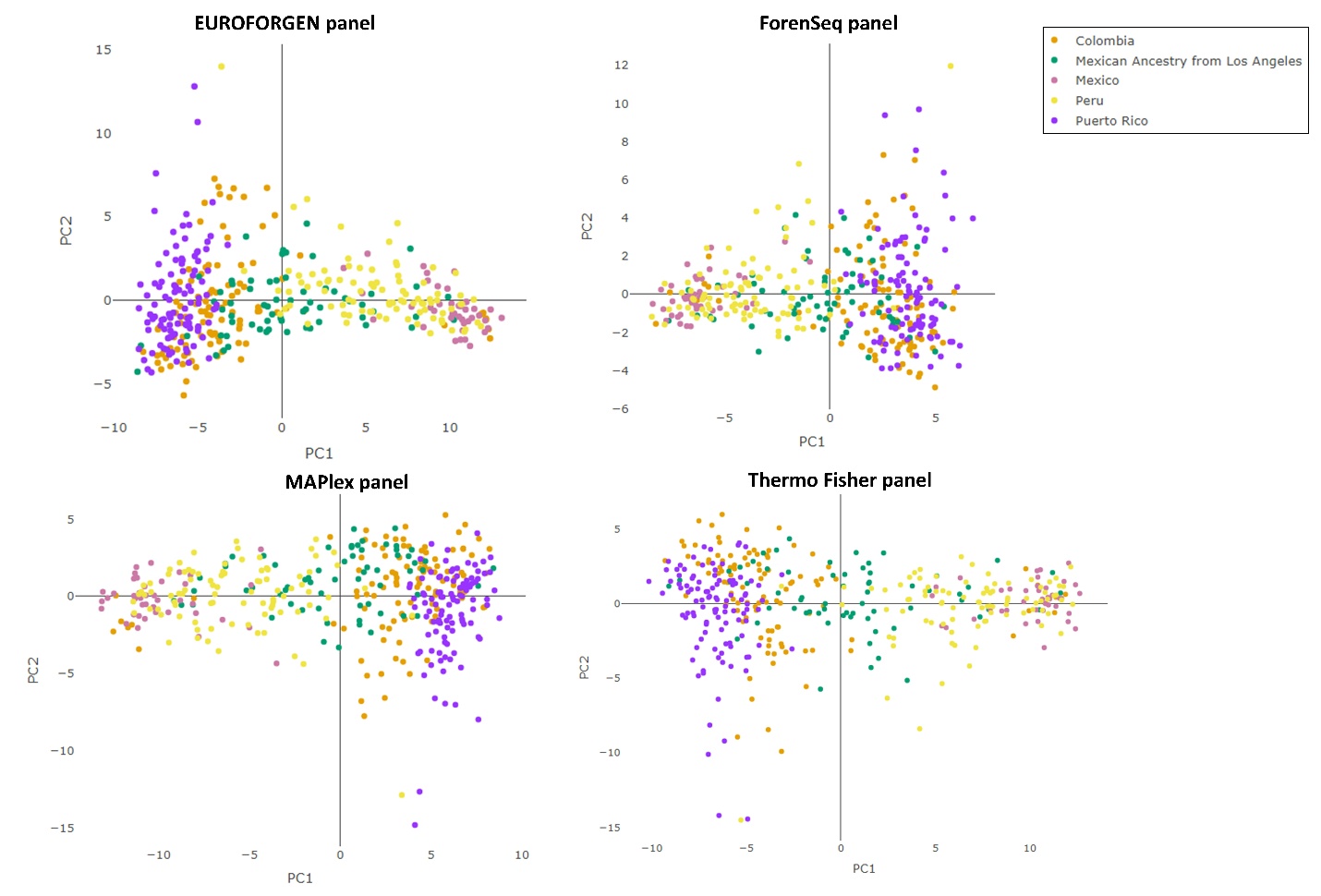


**Figure S.3:** PCA Scores plots showing the PCA models obtained for the American population using all forensic panels.


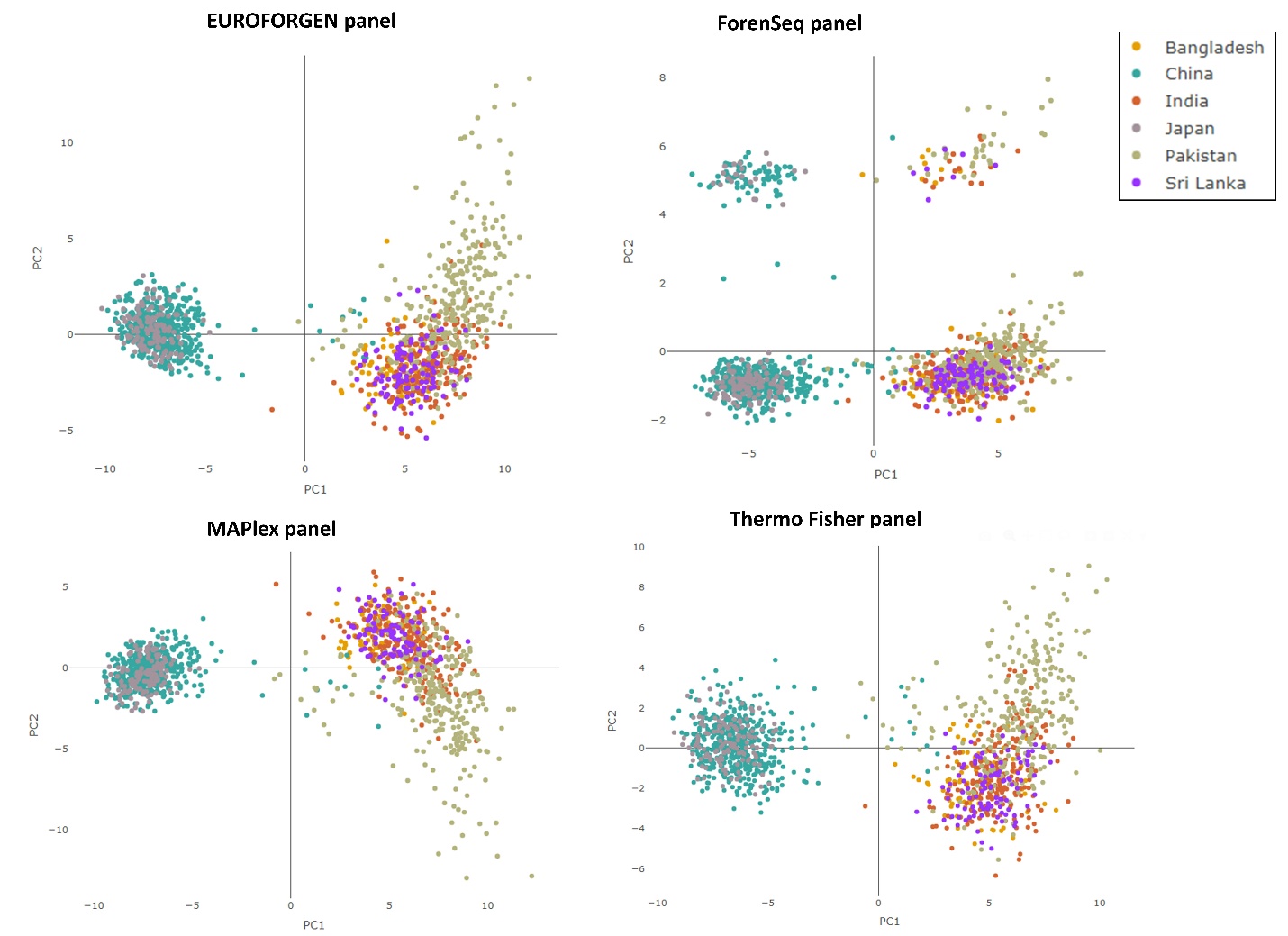


**Figure S.4:** PCA Scores plots showing the PCA models obtained for the Asian population using all forensic panels.


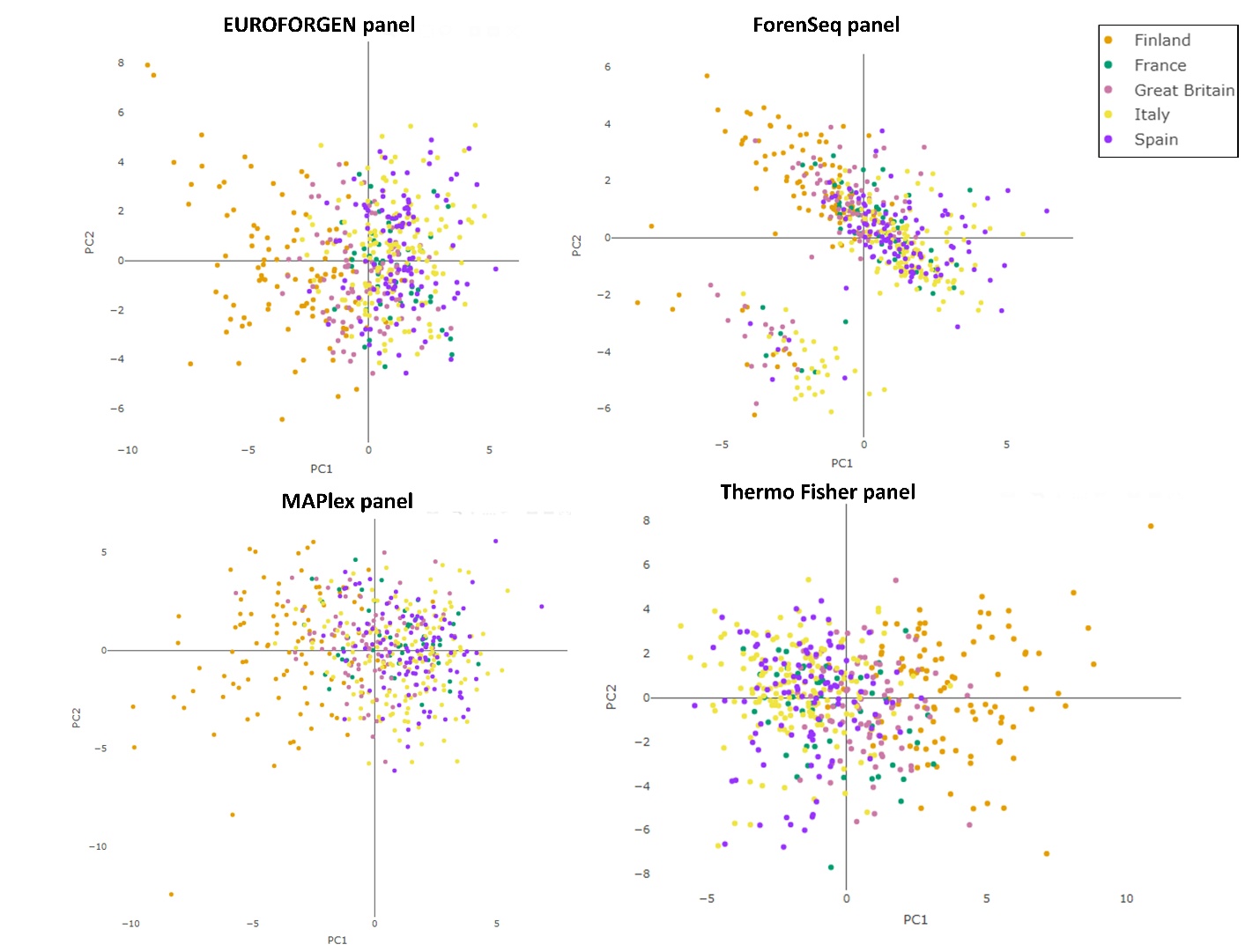


**Figure S.5:** PCA Scores plots showing the PCA models obtained for the European population using all forensic panels


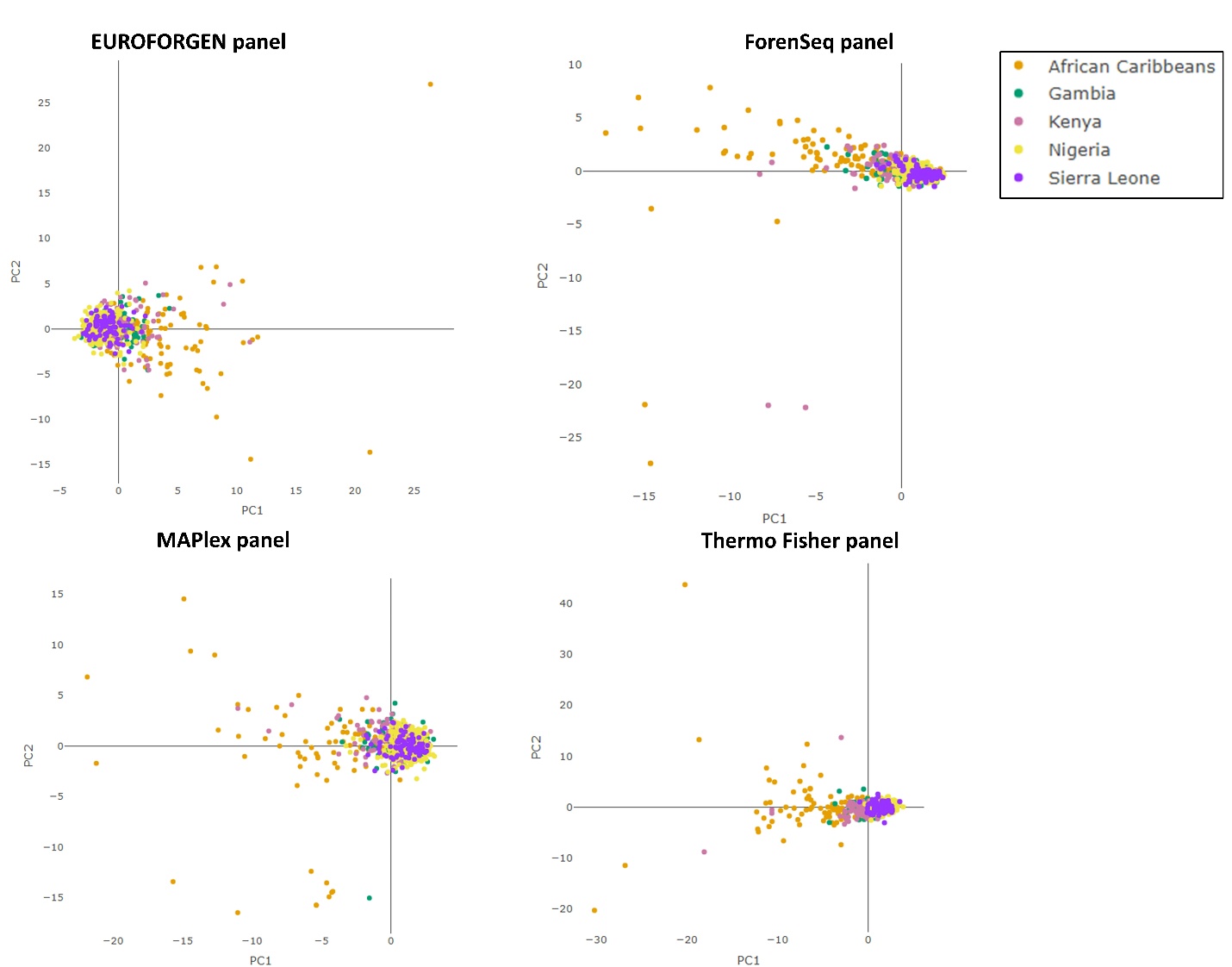


**Figure S.6:** Scores plots of the PLS-DA models for the African countries and populations.


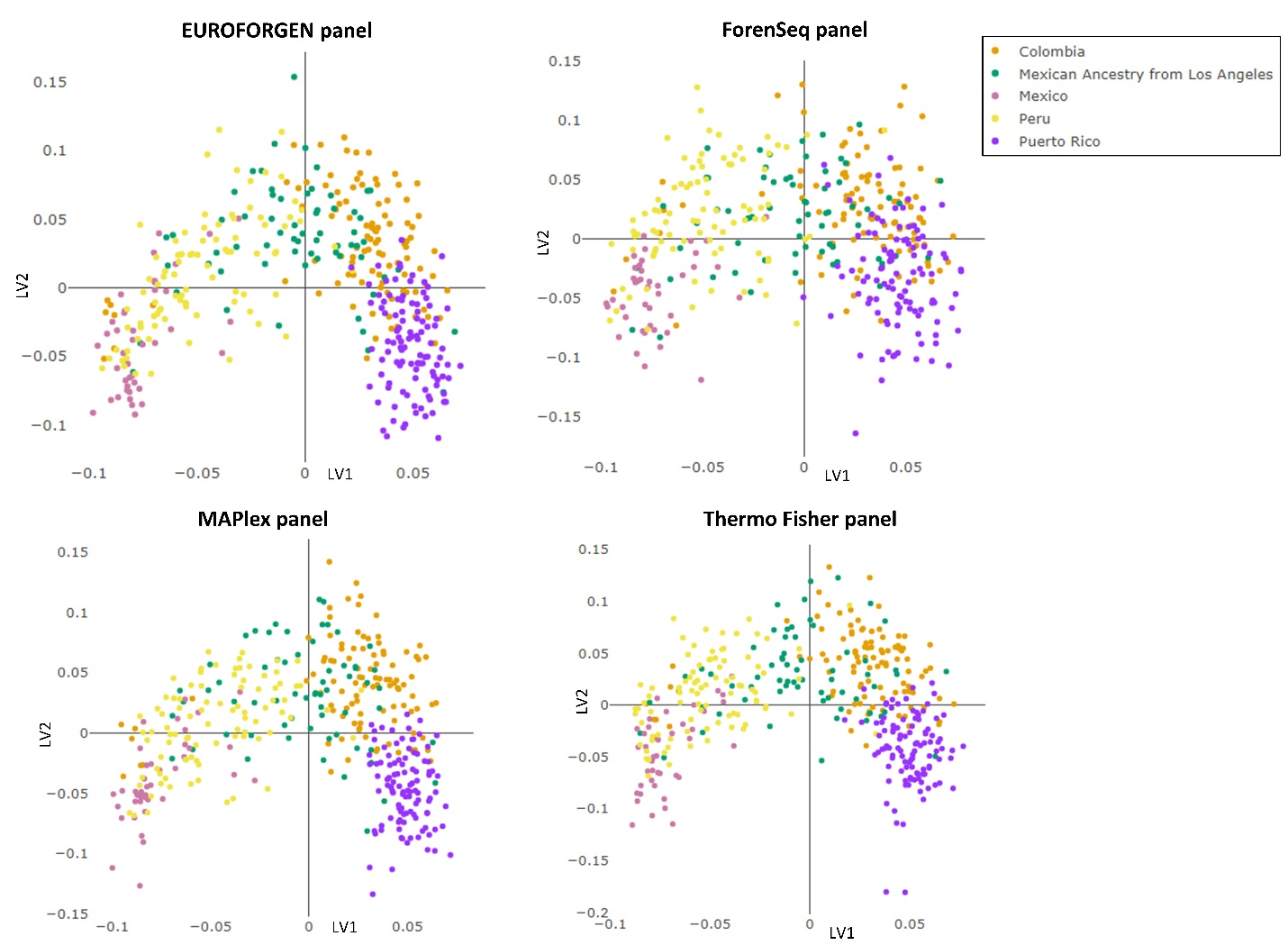


**Figure S.7:** Scores plots of the PLS-DA models for the American countries and populations.


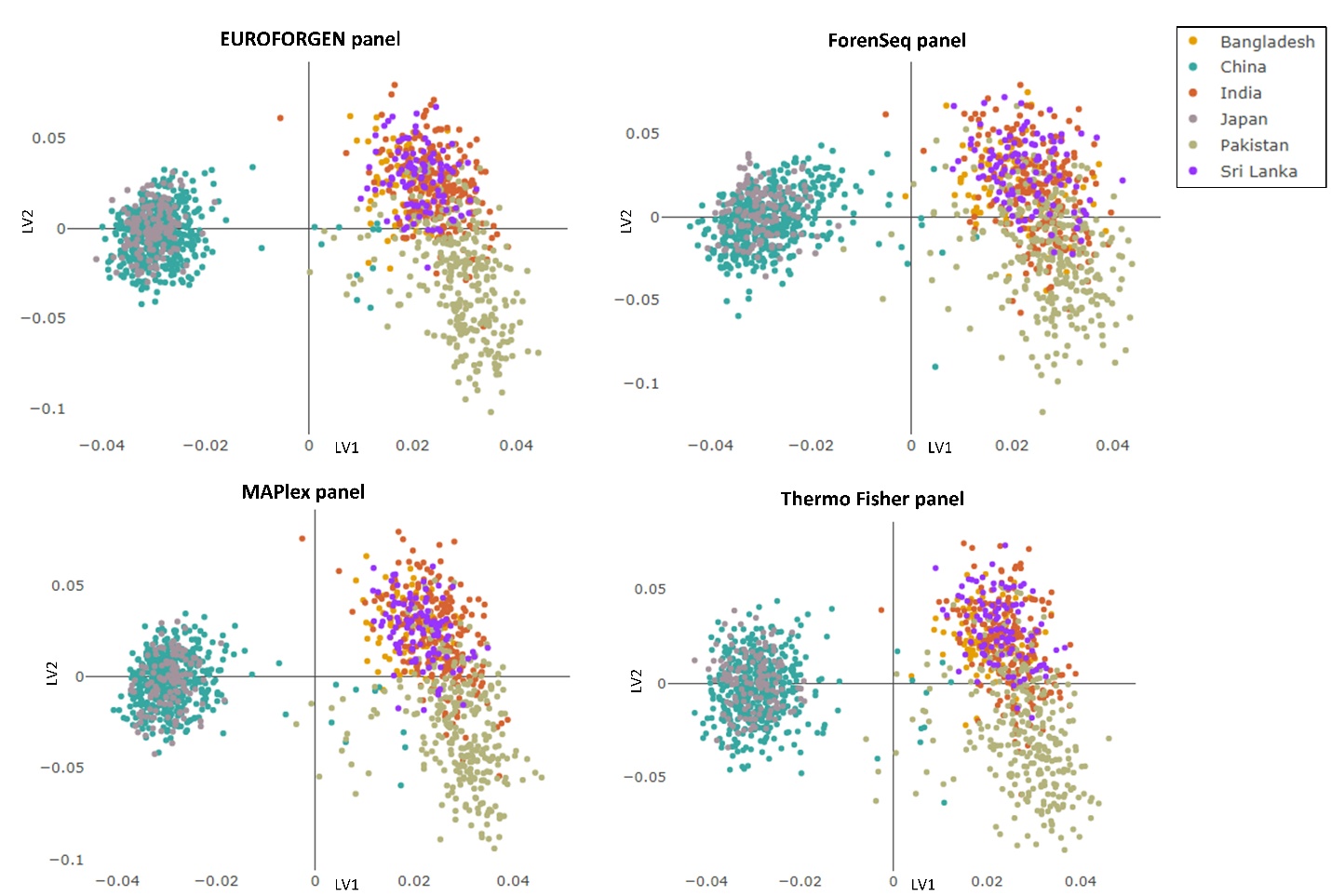


**Figure S.8:** Scores plots of the PLS-DA models for the Asian countries and populations.


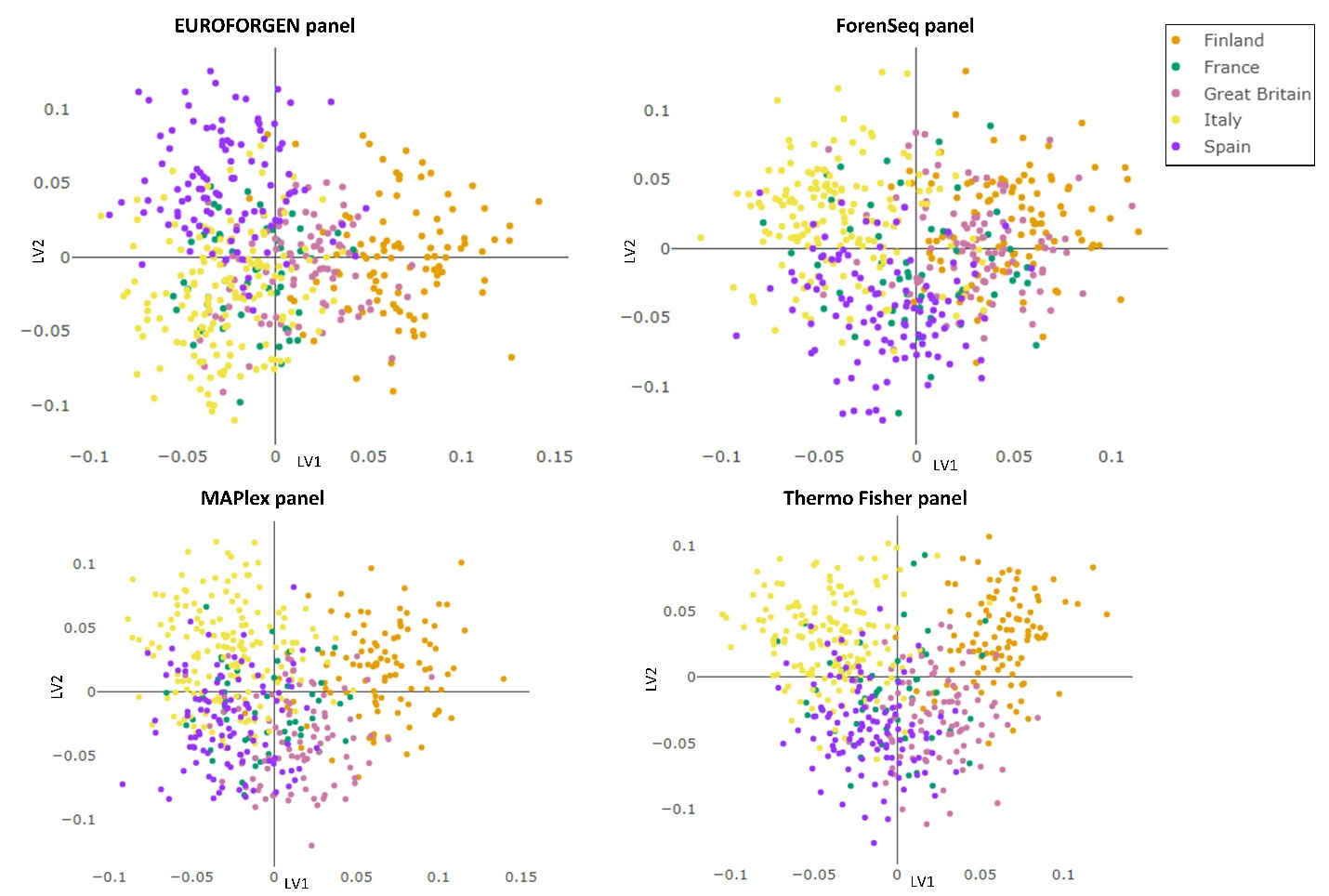


**Figure S.9:** Scores plots of the PLS-DA models for the European countries and populations.

**Supplementary Tables**

|  |  |  |  |
| --- | --- | --- | --- |
| **EUROFORGEN** | | | |
| **Populations** | **Sensitivity** | **Specificity** | **AUC** |
| African Caribbeans | 0.60 | 0.89 | 0.75 |
| Gambia | 0.66 | 0.89 | 0.77 |
| Kenya | 0.68 | 0.91 | 0.80 |
| Nigeria | 0.86 | 0.73 | 0.80 |
| Sierra Leone | 0.30 | 0.87 | 0.59 |
|  |  |  |  |
| **ForenSeq** | | | |
| **Populations** | **Sensitivity** | **Specificity** | **AUC** |
| African Caribbeans | 0.56 | 0.89 | 0.73 |
| Gambia | 0.68 | 0.86 | 0.77 |
| Kenya | 0.65 | 0.99 | 0.82 |
| Nigeria | 0.83 | 0.80 | 0.81 |
| Sierra Leone | 0.25 | 0.86 | 0.55 |
|  |  |  |  |
| **MAPlex** | | | |
| **Populations** | **Sensitivity** | **Specificity** | **AUC** |
| African Caribbeans | 0.59 | 0.91 | 0.75 |
| Gambia | 0.67 | 0.84 | 0.75 |
| Kenya | 0.68 | 0.95 | 0.81 |
| Nigeria | 0.79 | 0.78 | 0.78 |
| Sierra Leone | 0.31 | 0.90 | 0.60 |
|  |  |  |  |
| **Thermo Fisher** | | | |
| **Populations** | **Sensitivity** | **Specificity** | **AUC** |
| African Caribbeans | 0.63 | 0.96 | 0.79 |
| Gambia | 0.62 | 0.87 | 0.75 |
| Kenya | 0.69 | 0.90 | 0.80 |
| Nigeria | 0.88 | 0.82 | 0.85 |
| Sierra Leone | 0.25 | 0.86 | 0.55 |

**Table S.1:** Sensitivity, specificity, and AUC values of the optimal XGBoost model built for the African countries and populations.

| **EUROFORGEN** | | | |
| --- | --- | --- | --- |
| **Populations** | **Sensitivity** | **Specificity** | **AUC** |
| Colombia | 0.64 | 0.77 | 0.70 |
| Mexican Ancestry from Los Angeles | 0.37 | 0.91 | 0.64 |
| Mexico | 0.69 | 0.99 | 0.84 |
| Peru | 0.61 | 0.89 | 0.75 |
| Puerto Rico | 0.85 | 0.77 | 0.81 |
|  |  |  |  |
| **ForenSeq** | | | |
| **Populations** | **Sensitivity** | **Specificity** | **AUC** |
| Colombia | 0.67 | 0.80 | 0.73 |
| Mexican Ancestry from Los Angeles | 0.34 | 0.94 | 0.64 |
| Mexico | 0.69 | 0.98 | 0.83 |
| Peru | 0.68 | 0.89 | 0.79 |
| Puerto Rico | 0.77 | 0.79 | 0.78 |
|  |  |  |  |
| **MAPlex** | | | |
| **Populations** | **Sensitivity** | **Specificity** | **AUC** |
| Colombia | 0.68 | 0.73 | 0.70 |
| Mexican Ancestry from Los Angeles | 0.27 | 0.90 | 0.58 |
| Mexico | 0.64 | 0.99 | 0.81 |
| Peru | 0.70 | 0.93 | 0.81 |
| Puerto Rico | 0.85 | 0.82 | 0.83 |
|  |  |  |  |
| **Thermo Fisher** | | | |
| **Populations** | **Sensitivity** | **Specificity** | **AUC** |
| Colombia | 0.65 | 0.80 | 0.73 |
| Mexican Ancestry from Los Angeles | 0.37 | 0.91 | 0.64 |
| Mexico | 0.61 | 0.90 | 0.76 |
| Peru | 0.68 | 0.90 | 0.79 |
| Puerto Rico | 0.76 | 0.78 | 0.77 |

**Table S.2:** Sensitivity, specificity, and AUC values of the optimal XGBoost model built for the American countries and populations.

| **EUROFORGEN** | | | |
| --- | --- | --- | --- |
| **Populations** | **Sensitivity** | **Specificity** | **AUC** |
| Bangladesh | 0.11 | 0.95 | 0.53 |
| China | 0.91 | 0.89 | 0.90 |
| India | 0.67 | 0.83 | 0.75 |
| Japan | 0.66 | 0.94 | 0.80 |
| Pakistan | 0.82 | 0.90 | 0.86 |
| Sri Lanka | 0.45 | 0.85 | 0.65 |
|  |  |  |  |
| **ForenSeq** | | | |
| **Populations** | **Sensitivity** | **Specificity** | **AUC** |
| Bangladesh | 0.17 | 0.88 | 0.52 |
| China | 0.93 | 0.91 | 0.92 |
| India | 0.67 | 0.90 | 0.79 |
| Japan | 0.63 | 0.91 | 0.77 |
| Pakistan | 0.81 | 0.87 | 0.84 |
| Sri Lanka | 0.48 | 0.87 | 0.67 |
|  |  |  |  |
| **MAPlex** | | | |
| **Populations** | **Sensitivity** | **Specificity** | **AUC** |
| Bangladesh | 0.17 | 0.89 | 0.53 |
| China | 0.88 | 0.82 | 0.85 |
| India | 0.61 | 0.85 | 0.73 |
| Japan | 0.68 | 0.99 | 0.83 |
| Pakistan | 0.85 | 0.88 | 0.87 |
| Sri Lanka | 0.42 | 0.84 | 0.63 |
|  |  |  |  |
| **Thermo Fisher** | | | |
| **Populations** | **Sensitivity** | **Specificity** | **AUC** |
| Bangladesh | 0.13 | 0.89 | 0.51 |
| China | 0.92 | 0.89 | 0.91 |
| India | 0.65 | 0.89 | 0.77 |
| Japan | 0.63 | 0.90 | 0.76 |
| Pakistan | 0.76 | 0.86 | 0.81 |
| Sri Lanka | 0.50 | 0.89 | 0.69 |

**Table S.3:** Sensitivity, specificity, and AUC values of the optimal XGBoost model built for the Asian countries and populations.

| **EUROFORGEN** | | | |
| --- | --- | --- | --- |
| **Populations** | **Sensitivity** | **Specificity** | **AUC** |
| Finland | 0.63 | 0.90 | 0.76 |
| France | 0.75 | 0.93 | 0.84 |
| Great Britain | 0.28 | 0.90 | 0.59 |
| Italy | 0.59 | 0.89 | 0.74 |
| Spain | 0.77 | 0.92 | 0.85 |
|  |  |  |  |
| **ForenSeq** | | | |
| **Populations** | **Sensitivity** | **Specificity** | **AUC** |
| Finland | 0.69 | 0.85 | 0.77 |
| France | 0.75 | 0.92 | 0.83 |
| Great Britain | 0.27 | 0.87 | 0.57 |
| Italy | 0.62 | 0.85 | 0.73 |
| Spain | 0.70 | 0.96 | 0.83 |
|  |  |  |  |
| **MAPlex** | | | |
| **Populations** | **Sensitivity** | **Specificity** | **AUC** |
| Finland | 0.65 | 0.82 | 0.74 |
| France | 0.78 | 0.92 | 0.85 |
| Great Britain | 0.27 | 0.95 | 0.61 |
| Italy | 0.57 | 0.86 | 0.71 |
| Spain | 0.75 | 0.92 | 0.83 |
|  |  |  |  |
| **Thermo Fisher** | | | |
| **Populations** | **Sensitivity** | **Specificity** | **AUC** |
| Finland | 0.65 | 0.86 | 0.76 |
| France | 0.78 | 0.96 | 0.87 |
| Great Britain | 0.27 | 0.86 | 0.56 |
| Italy | 0.67 | 0.87 | 0.77 |
| Spain | 0.74 | 0.92 | 0.83 |

**Table S.4**: Sensitivity, specificity, and AUC values of the optimal XGBoost model built for the European countries and populations.
